# Bowel burdens: a systematic review and meta-analysis examining the relationships between bowel dysfunction and quality of life after spinal cord injury

**DOI:** 10.1038/s41393-024-01002-8

**Published:** 2024-07-16

**Authors:** Elin K. Sober-Williams, Rebekah H. Y. Lee, David G. T. Whitehurst, Christopher B. McBride, Rhonda Willms, Victoria E. Claydon

**Affiliations:** 1 Department of Biomedical Physiology and Kinesiology, Simon Fraser University, Burnaby, BC Canada; 2 International Collaboration On Repair Discoveries (ICORD), University of British Columbia, Vancouver, BC Canada; 3 Faculty of Health Sciences, Simon Fraser University, Burnaby, BC Canada; 4 Spinal Cord Injury British Columbia, Vancouver, BC Canada; 5 GF Strong Rehabilitation Centre, Spinal Cord Injury Program, Vancouver Coastal Health, Vancouver, BC Canada; 6 Division of Physical Medicine and Rehabilitation, Faculty of Medicine, University of British Columbia, Vancouver, BC Canada

**Keywords:** Quality of life, Digestive signs and symptoms

## Abstract

**Study design:**

Systematic review and meta-analysis.

**Objectives:**

Many individuals with spinal cord injury (SCI) experience autonomic dysfunction, including profound impairments to bowel and cardiovascular function. Neurogenic bowel dysfunction (NBD) is emerging as a potential determinant of quality of life (QoL) after SCI. For individuals with high-level lesions ( > T6), bowel care-related autonomic dysreflexia (B-AD; profound episodic hypertension) further complicates bowel care. We aimed to evaluate the extent of bowel dysfunction after SCI, and the impact of bowel dysfunction on QoL after SCI.

**Methods:**

We searched five databases to identify research assessing the influence of NBD or B-AD on QoL after SCI. Metrics of bowel dysfunction (fecal incontinence [FI], constipation, time to complete, and B-AD) and QoL data were extracted and synthesised. Where possible, meta-analyses were performed.

**Results:**

Our search identified 2042 titles, of which 39 met our inclusion criteria. Individuals with SCI identified problems with NBD (74.7%), FI (56.9%), and constipation (54.6%), and 49.3% of individuals with SCI > T6 experienced B-AD. Additionally, 40.3% of individuals experienced prolonged defecation ( > 30 min). Moderate/severe deterioration in QoL due to NBD was reported by 55.5% of individuals with SCI, with negative impacts on physical, emotional, and social health-related QoL associated with inflexibility of bowel routines, fear of accidents, and loss of independence.

**Conclusion:**

Bowel dysfunction and bowel care challenges are prevalent and disabling for individuals with SCI, with a profoundly negative impact on QoL. Improving bowel management is a key target to improve QoL for those living with SCI.

## Introduction

Over 296,000 Americans [1] and 86,000 Canadians [2] live with a spinal cord injury (SCI). In addition to motor and sensory deficits, SCI can also disrupt descending autonomic pathways, with profound consequences to autonomic processes including cardiovascular and bowel function. Neurogenic bowel dysfunction (NBD) after SCI can be complicated by non-neurogenic factors, and often presents with constipation and fecal incontinence (FI). While NBD can be managed with carefully planned bowel routines, these programmes may be inflexible and time consuming, imposing limitations to lifestyle and participation in activities away from home [3, 4]. NBD is a major life-limiting problem [5], and its impact is reported to exceed other autonomic and motor deficits [3, 6]. Indeed, bowel management is a potential determinant of quality of life (QoL) for those with SCI [7].

While normal bowel emptying can be compromised at all lesion levels, patterns of NBD differ according to lesion level. Individuals with upper motor neuron bowel dysfunction (SCI at or above T12) typically experience reflexive NBD [8], where excessive muscle tone in the colon wall and anal sphincters results in increased colonic transit time and constipation [9]. The associated inability to voluntarily relax the anal sphincter necessitates chemical or mechanical stimuli to trigger reflex defecation [8]. Individuals with lower motor neuron injuries (below T12) typically experience areflexic NBD characterised by reduced peristalsis, coupled with reduced colonic and sphincter tone. Consequently, individuals with an areflexic bowel experience constipation, but are also at an increased risk of FI as the external anal sphincters are relaxed [8].

For individuals with high-level injuries (at or above the level of sympathetic innervation (T6) to the splanchnic vasculature), autonomic dysreflexia (AD) is a cardiovascular complication that often intersects with bowel function [10]. AD refers to profound hypertension secondary to sensory stimuli below the lesion [3, 11]. Bowel impaction and bowel management techniques are among the most potent stimuli for AD [12]. Episodes of AD are associated with high morbidity and mortality [13–15], are often highly symptomatic [16, 17], and can occur multiple times per day [18], which can profoundly interfere with activities of daily living and QoL [3, 6, 19].

Given the complexity of bowel care challenges, individuals with SCI establish routines to regulate their bowel movements and manage dysfunction. These routines require planning, substantial time to complete, and involve both bowel care (the process of bowel emptying, often involving assistive techniques such as digital rectal stimulation or manual evacuation) and a surrounding bowel program (consideration of diet and fluid intake, activity, medications, and assistive devices) [20]. Due to impaired mobility and hand function, personal assistance may be required to complete bowel care. Regular scheduling is critical [20], which impacts QoL by limiting flexibility, the ability to work away from home, and participation in social activities [3, 5, 6], leading to feelings of frustration and discontent [21]. Simply stated, “the bowel rules” [20].

While existing reviews explore bowel problems in the context of specific management techniques [22–24], none have systematically evaluated the full landscape of NBD post-SCI and the ongoing bowel care-related challenges faced by individuals with SCI, including FI, constipation, bowel care-related AD (B-AD), and the burden of care routines. In this community-partnered study, we conducted a systematic review to examine *quantitative* (collective data summaries) and *qualitative* (perspectives of those with lived experience in their own narrative) evidence regarding: (i) the extent of bowel dysfunction after SCI, as defined by the timing of bowel emptying, presence of FI and constipation, and the prevalence of B-AD; and (ii) the impact of bowel dysfunction on QoL after SCI.

## Methods

This systematic review and meta-analysis followed the Cochrane Handbook for Systematic Reviews [25] and the Preferred Reporting Items for Systematic Reviews and Meta-Analyses (PRISMA) [26] guidelines.

### Databases and search strategy

A literature search across five databases (PubMed, Web of Science, CINAHL, PsycINFO, and Embase) was performed (September 7th 2022). Search terms were developed through a literature search, MeSH term search, and in consultation with a research librarian who specializes in conducting systematic reviews and meta-analyses. The final keyword list and search strategy comprised terms for QoL and bowel dysfunction or B-AD after SCI (Supplemental Table 1). Articles identified from database searches were compiled using Zotero (version 5.0.96.1).

### Eligibility criteria and study selection

Articles were evaluated for eligibility first through title and abstract screening, then through full text screening. Both steps were completed by two independent reviewers (EKSW and RHYL). Articles were included if they were peer-reviewed, available online, included data on participants aged 18 years and older with SCI (traumatic or non-traumatic, any level or severity of injury, and injury duration of at least six months at time of study), included data on NBD and/or B-AD, and evaluated impacts of these conditions on QoL. We excluded case reports, case series, grey literature, or articles about a non-human or non-SCI population. Articles written in languages other than English were translated for evaluation. In cases of disagreement between the two reviewers, an expert in the field (VEC) helped establish a consensus. The reasons for excluding full-text articles were recorded.

### Data extraction

Data extraction from the included articles was completed independently by two reviewers (EKSW and RHYL) in duplicate, and in the case of any disagreement, an expert in the field (VEC) was consulted. Where available, data extraction included publication details, study design, study primary and secondary outcomes, population characteristics (traumatic/non-traumatic SCI, sample size, sex, age, and injury duration), and injury characteristics (lesion level and severity of motor and sensory impairment). QoL instruments and scores were extracted, along with measures of bowel function: frequency of bowel emptying, time to complete bowel care, and whether bowel routines were successful, as evaluated by the severity and frequency of constipation and incontinence episodes.

### Quality evaluation

Quality evaluation of articles selected for inclusion in the analyses was performed using the Quality Assessment with Diverse Studies (QuADS) tool [27]. This tool is a revised and enhanced version of the Quality Assessment Tool for Studies with Diverse Designs (QATSDD) [28] that enhances its applicability for use in health research, and particularly for systematic reviews that include diverse study designs and mixed or multi-method studies. It has high inter-rater reliability and content and face validity [27]. As recommended in the scoring guidelines [27] three members of the study team (VEC, RHYL, EKSW) first confirmed the appropriateness of the tool for the present study, agreed upon how to apply the criteria in the context of the work, and independently calibrated the scoring on three randomly selected articles. Once scoring guidelines and consensus were met, the same three team members independently applied the criteria to the remainder of the studies according to the agreed upon approach. In cases of uncertainty or ambiguity in scoring, group consensus was sought to arrive at a final score. All articles were scored based on 13 metrics of quality, with a range for each item of 0 (poor quality) to 3 (high quality), comprising a total score ranging from 0-39. Note that there is no cutoff total score for consideration of high or low quality, rather criteria are intended to be comprehensively and narratively evaluated [27].

### Analytic considerations

For time to complete care, routines lasting more than 30 min were considered prolonged [3, 29, 30], and more than 60 minutes clinically problematic [20]. Data are reported as means and standard deviations. Where descriptive data were originally reported using other statistics (e.g., median, range, interquartile range, or confidence interval), means and standard deviations were calculated or estimated [31]. Summary component scores of the 36-item Short Form health survey (SF-36) were calculated from the domain score means and standard deviations, where necessary [32]. Where data were presented only in figures, means and standard deviations were extracted using WebPlotDigitizer (version 4.4; Pacifica, Ca, USA). Analyses were completed in Microsoft Excel (version 16.62). Data visualisation was completed in SigmaPlot (version 14.0) and Microsoft PowerPoint (version 16.64).

Where possible, meta-analyses were performed. Results from questions with comparable language and structure were aggregated as weighted averages ($\bar{x}$) and reported with the combined sample size. Where studies reported data from two subgroups without indication of statistical significance, unpaired t-tests were performed based on means, standard deviations, and sample size. Aggregate metrics of bowel dysfunction, FI, and time to complete were combined and reported as weighted average ($\bar{x}$) proportions. A summary estimate of effect for the influence of bowel dysfunction on QoL and satisfaction with bowel care was calculated and visualised using forest plots. Relationships between severity of bowel dysfunction and SF-36 summary component scores were examined by linear regression with Pearson or Spearman correlations, as appropriate.

A three-stage structured analysis adapted from Larsen et al. (2022) was applied to analyse findings from qualitative studies [33]. First, we identified units of meaning (relevant quotes from individuals with SCI that addressed our research questions), then units of significance (the meaning of the quote as defined through a dynamic reading process by two independent reviewers [EKSW and RHYL]), and through interpretation, the overarching theme [33]. All three stages were subject to critical interpretation and discussion by the authors who considered the information in context with the available literature and their own understandings of theoretical concepts [33].

### Knowledge partners

To ensure our findings were contextualised to the lived experience of individuals living with SCI and their caregivers, we included local SCI clinicians and an SCI community partner agency (Spinal Cord Injury British Columbia) as knowledge partners in the research. These research users provided critical feedback, insight, and context to the conclusions drawn in the study.

## Results

**Fig. 1:**
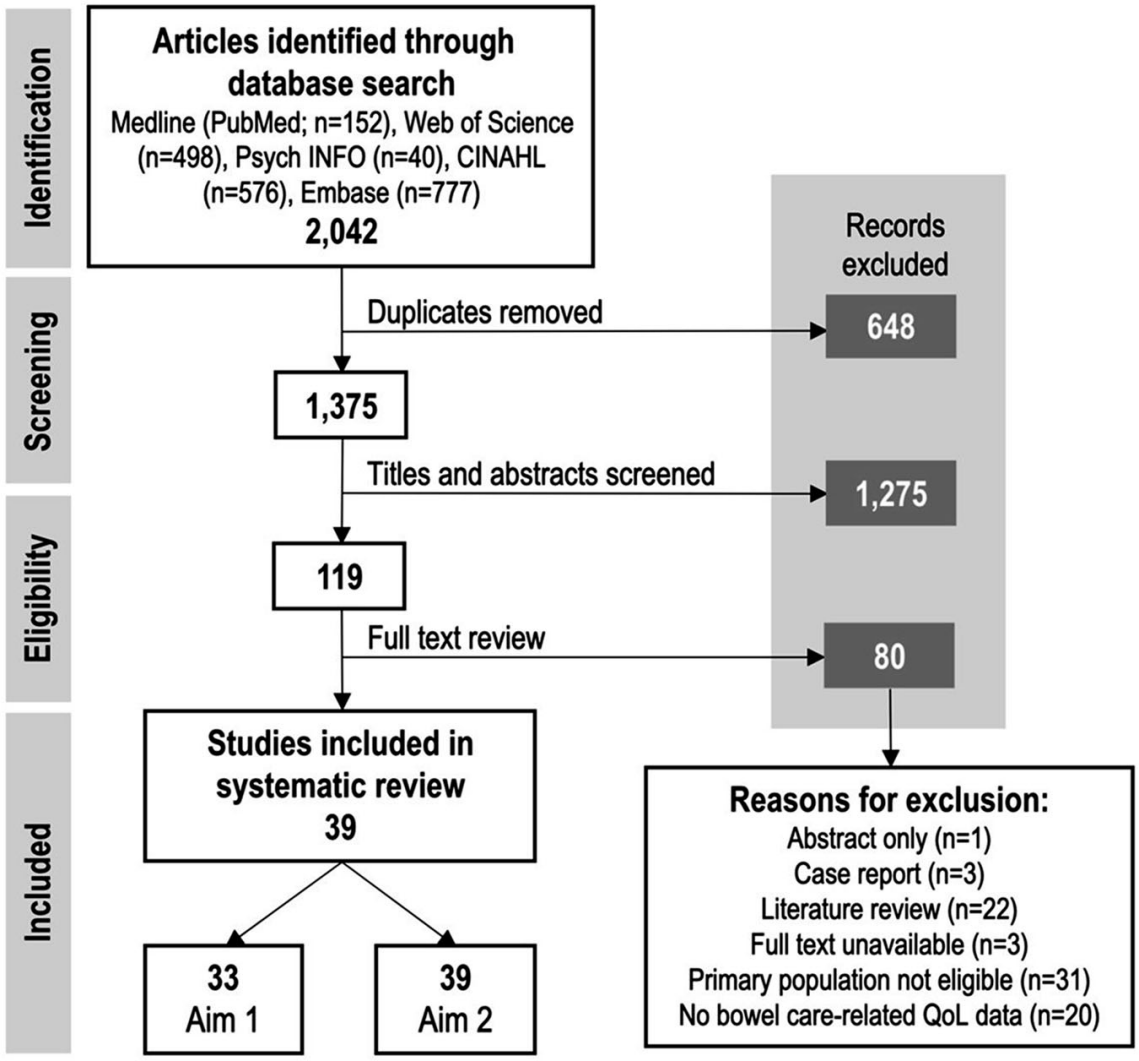
PRISMA diagram summarising the study selection process [26]. The search identified 2,042 articles. After screening and eligibility review, 39 articles met our inclusion criteria.

Our search identified 2042 articles. After duplicate removal and screening, 39 articles met our inclusion criteria (Fig. 1). The study characteristics for the included articles are summarised in Table 1. Three papers used interview data, providing a qualitative perspective to the research question. Supplemental Table 2 provides a summary of the outcome measures and instruments used.

**Table 1:** Study characteristics.

Study	Traumatic SCI (%)	Sample size (female)	Age (years)	Duration	Injury level (%)	Tetraplegia/Paraplegia (%)	AIS grade (A/B/C/D/E) (%)	AIS complete vs incomplete (%)
Adriaansen et al. 2016 [49]	91	282 (73)	48^a^	Chronic	⎯	41/59	68/14/10/8/0	68/32
Burns et al. 2015 [21]	⎯	19 (6)	42 (13)	Chronic	⎯	53/47	⎯	74/26
Carlozzi et al. 2013 [19]	78	27 (3)	66 (13)	Chronic	⎯	44/56	-	59/41
Coggrave et al. 2009 [6]	⎯	1334 (355)	52^a^	Chronic	C:41; T: 50; L: 8; S: 1^b^	⎯	⎯	48/43^b^
Coggrave et al. 2012 [59]	⎯	92 (28)	56 (9)	Chronic	C: 28; T: 66; L: 4^b^	⎯	⎯	70/25^b^
Craven et al. 2012 [35]	78	357 (61)	54	Chronic	⎯	52/48	⎯	40/60
Elmelund et al. 2019 [48]	28	684 (684)	55 (20)	⎯	C: 33; T: 25; L: 11; S: 2^b^	⎯	7/4/11/77/1	7/93
Erdem et al. 2017 [43]	95	42 (8)	⎯	Chronic	C: 29; T: 57; L: 14	⎯	55/10/26/10/0	55/45
Gong et al. 2021 [39]	83	101 (26)	41 (15)	⎯	C: 29; T: 49; L: 9; S: 13	⎯	⎯	40/60
Gorman et al. 2021 [60]	⎯	49 (11)	39 (14)	Chronic	⎯	28/72	A/B: 62; C/D: 38	⎯
Hicken et al. 2001 [64]	81	106 (8)	36.9	Chronic	⎯	91/9	⎯	9/91
Hwang et al. 2017 [51]	90^b^	131 (47)	⎯	Chronic	⎯	59/40	⎯	76/24
Inskip et al. 2018 [3]	⎯	287	49 (13)	Chronic	C: 45; T: 45; L: 9; S: 1	⎯	⎯	30/70
Jorgensen et al. 2017	62	123 (36)	63 (9)	Chronic	⎯	⎯	⎯	⎯
Jorgensen et al. 2021	64	78 (25)	68 (8)	Chronic	⎯	⎯	⎯	⎯
Kannisto & Rintala 1995 [47]	⎯	35 (10)	31^a^	Chronic	C: 23; T: 77	⎯	⎯	77/23
Kim et al. 2012 [45]	93	388 (94)	45 (11)	Chronic	⎯	31/62^b^	⎯	54/40^b^
Koyuncu et al. 2017 [40]	91	55 (13)	36 (12)	Mixed^c^	C: 18; T: 51; L: 31	⎯	44/20/15/22/0	44/56
Krogh et al. 1997 [52]	83	424 (124)	41	Chronic	C: 41; T: 37; L: 19^b^	⎯	⎯	60/40
Krogh et al. 2006 [41]	75	424 (124)	41	Chronic	C: 43; T: 38; L: 19	⎯	⎯	60/40
Leduc et al. 2002 [29]	100	30 (4)	40	Chronic	⎯	50/50	70/30/0/0/0	70/30
Liu et al. 2009 [42]	88	128 (33)	48	Chronic	⎯	28/45^b^	0/0/73/27/0	0/100
Locke et al. 2019 [53]	72	1529 (500)	50 (14)	Chronic	⎯	24/70^b^	⎯	⎯
Lombardi et al. 2010 [54]	78	23 (8)	36 (9)	⎯	⎯	⎯	0/0/39/61/0	0/100
Luther et al. 2005 [62]	⎯	296 (3)	⎯	Chronic	⎯	⎯	⎯	⎯
Lynch et al. 2000 [55]	⎯	467 (83)	44	Chronic	C: 50; T: 31; L: 19	⎯	⎯	50/50
Mazor et al. 2016 [56]	81	21 (17)	50	Chronic	⎯	⎯	⎯	0/100
McCarthy et al. 2020 [46]	⎯	50	⎯	Chronic	⎯	⎯	⎯	⎯
Nevedal et al. 2016 [63]	94	50 (50)	46 (15)	Chronic	⎯	58/42	⎯	46/54
Nielsen et al. 2017 [58]	69	109 (26)	55	Chronic	C: 9; T: 30; L: 61	⎯	⎯	66/34
Noonan et al. 2008 [34]	100	70 (13)	51 (18)	Chronic	C: 100	⎯	⎯	⎯
Pardee et al. 2012 [30]	⎯	241	⎯	⎯	⎯	⎯	⎯	⎯
Pires et al. 2018 [38]	72	64 (22)	57 (16)	Chronic	C: 39; T: 39; L: 22	⎯	39/13/17/31/0	39/61
Smith & Decter 2015 [65]	⎯	17 (5)	33	Chronic	C: 47; T: 53	⎯	⎯	⎯
Stoffel et al. 2021 [61]	⎯	1373 (546)	44 (13)	Chronic	C: 44; T: 47; L/S:9	⎯	26/16/11/3/0^b^	26/31^b^
Sweet et al. 2014	100	1137 (508)	48 (13)	Chronic	⎯	50/50	38/8//20/21/2^b^	38/51^b^
Ture et al. 2022 [44]	100	92 (19)	45 (15)	Chronic	C: 20; T: 47; L: 34	⎯	46/13/11/30/0	46/54
Van Doorn et al. 2022 [57]	⎯	55 (19)	54 (16)	Chronic	C: 26; T: 53; L:16; ^b^	⎯	27/13/18/16/2^b^	27/49^b^
Westgren & Levi 1998 [36]	100	320 (59)	42	⎯	⎯	39/55^b^	⎯	⎯

Where applicable, data are presented as mean (standard deviation) unless otherwise stated or incomplete data were provided. The injury duration was considered chronic if the SCI was sustained greater than 6 months previously and the participants had been discharged from hospital/in-patient rehabilitation. Injury levels are reported as percentages with cervical (C), thoracic (T), lumbar (L) or sacral (S) SCI. Where available the percentage of individuals with paraplegia and tetraplegia are provided. American Spinal Injury Association Impairment Scale (AIS) grades are provided where possible, with the percentage of individuals with a motor and sensory complete lesion (AIS A) and incomplete (AIS B, C, D, E) indicated.

^a^denotes only median age provided.

^b^denotes some participants listed as “unknown” for this category.

^c^denotes a mixed population with 47% chronic SCI, 29% subacute SCI (3–12 months) and 24% acute SCI (0–3 months).

Average total article quality scores based on the QUADS rating scale were 32.3 ± 0.2 (range 22–38). Individual item scores were high for most aspects under consideration, with average scores for 11 of the 13 items being appraised as 2.5 or greater. Individual items scores were poor for two aspects: (i) consideration of stakeholders in the research design or conduct (0.7 ± 0.1) and (ii) critical discussion of strengths and limitations (2.0 ± 0.2).

### AIM 1: efficacy of bowel routines and extent of bowel dysfunction

**Fig. 2:**
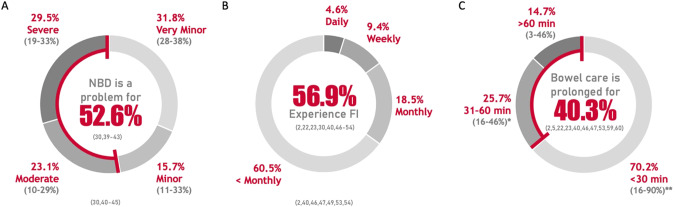
Severity of neurogenic bowel dysfunction, frequency of fecal incontinence, and time to complete bowel care in individuals with SCI. **A** Across the seven studies (*n* = 901) that used the Neurogenic Bowel Dysfunction (NBD) score unmodified, NBD was problematic for most individuals living with SCI (52.6%). **B** A weighted average of 56.9% of individuals in 14 studies (n = 4111) experienced fecal incontinence (FI). Seven studies (n = 2010) reported the frequency with which individuals with SCI experienced FI: 4.6% experienced FI daily; 9.4% weekly; 18.5% monthly; and 60.5% less than monthly. **C** In the self-reported average time to complete bowel care from 6 studies, a weighted average of 70.2% of participants required 30 minutes or less, 25.7% required 31–60 min, and 14.7% required over 60 min. Grey percentages indicate the range of responses in different studies. Red percentages indicate the weighted average. * Indicates data from references [3, 38, 45, 46, 52]; ** indicates data from references [3, 6, 29, 30, 38, 45, 46, 52, 59, 60].

Thirty-three papers addressed Aim 1 (Supplemental Table 3). Bowel function was measured through seven established measures. Sixteen studies used non-established (i.e., study-specific) questionnaires to assess bowel function. A wide range (15–99%; $\bar{x}$ = 74.7% [n = 2129]) of participants across five studies self-reported problems with bowel dysfunction [6, 34–37]. Across seven studies [n = 901] that used the NBD score in an unmodified format, the NBD severity distribution was: “Very minor” for 28–38% ($\bar{x}$ = 31.8%), “Minor” for 11–33% ($\bar{x}$ = 15.7%), “Moderate” for 10-29% ($\bar{x}$ = 23.1%), and “Severe” for 19–33% ($\bar{x}$ = 29.5%) of participants [38–44] (Fig. 2A).

### Fecal incontinence

FI was evaluated in 21 studies. In 14 studies, 12–100% ($\bar{x}$ = 56.9% [n = 4111]) of participants reported experiencing FI [3, 29, 30, 38, 39, 45–53] (Fig. 2B). In the seven studies (n = 2010) that examined the frequency of FI; 2-11% ($\bar{x}$ = 4.6%) experienced FI daily, 4–26% ($\bar{x}$ = 9.4%) weekly, 13-26% ($\bar{x}$ = 18.5%) monthly, and 40-75% (60.5%) less than monthly [3, 38, 45, 46, 48, 52, 53] (Fig. 2B). One study demonstrated that FI occurred more frequently in individuals with a lower level of SCI [3]. Severity of FI was indicated by the Wexner Score with scores of 3.6 and 16.7 ($\bar{x}$ = 4.3 [n = 495]) [54, 55], and the FISI with scores of 23 and 28 ($\bar{x}$ = 26.6 [n = 76]) [56, 57]. Higher scores indicate greater frequency of symptoms and symptom-related disruptions.

### Constipation

Considering the prevalence of constipation, seven studies found that 11–81% ($\bar{x}$ = 54.6% [n = 3551]) of participants self-reported problems with constipation [6, 30, 49, 50, 53, 56, 58]. In three studies, 49-54% ($\bar{x}$ = 52.4% [n = 88]) of participants had prolonged transit time [29, 47, 54].

### Frequency and time to complete bowel care

Eight studies considered the frequency with which individuals with SCI complete bowel care. These studies showed that 9–51% ($\bar{x}$ = 34.7% [n = 943]) of participants performed bowel care daily [3, 30, 38, 45, 46], 48–91% ($\bar{x}$ = 62.2% [n = 763]) one to six times per week [3, 38, 45–47], and 0–13% ($\bar{x}$ = 7.3% [n = 728]) less than once per week [3, 38, 45, 46]. Thirteen studies evaluated the time to complete bowel care (Fig. 2C). The self-reported average time to complete was 30 min or less for 16-90% ($\bar{x}$ = 70.2% [n = 1931]) of participants [3, 38, 45, 46, 52, 59], 31–60 min for 16–46% ($\bar{x}$ = 25.7% [n = 1237]) of participants [3, 38, 45, 46, 52], and 3–46% ($\bar{x}$ = 14.7% [n = 2751]) required more than 60 minutes [3, 6, 29, 30, 38, 45, 46, 52, 59, 60].

### Frequency of B-AD

Two studies found 45–74% ($\bar{x}$ = 49.3% [n = 1144]) of those at risk for AD (injury above T6) experienced AD during bowel care [3, 6]. When considering individuals with all lesion levels, six studies report 13–42% ($\bar{x}$ = 29.3% [n = 1068]) of all respondents experienced AD or symptoms compatible with AD during bowel care [30, 38, 45, 46, 52]. Using the Autonomic Dysfunction Following Spinal Cord Injury (ADFSCI) questionnaire, one study found that ADFSCI scores (corresponding to the frequency and severity of AD) were positively associated with NBD severity (p = 0.02) [61].

### AIM 2: the impact of bowel dysfunction on QoL

Thirty-nine papers addressed Aim 2. The impact of bowel care on QoL was considered using bowel care-specific QoL measures in 19 studies (Supplemental Table 4). Of these, established bowel care-specific instruments were used in six papers, while 13 studies used self-drafted bowel care-specific QoL questionnaires. Three studies conducted interviews and extracted themes using an established International Classification of Functioning, Disability, and Health framework. Additionally, 18 studies reported statistical associations between general (i.e., not bowel care-specific) measures of QoL and bowel management.

### Overall impact of bowel dysfunction on QoL

**Fig. 3:**
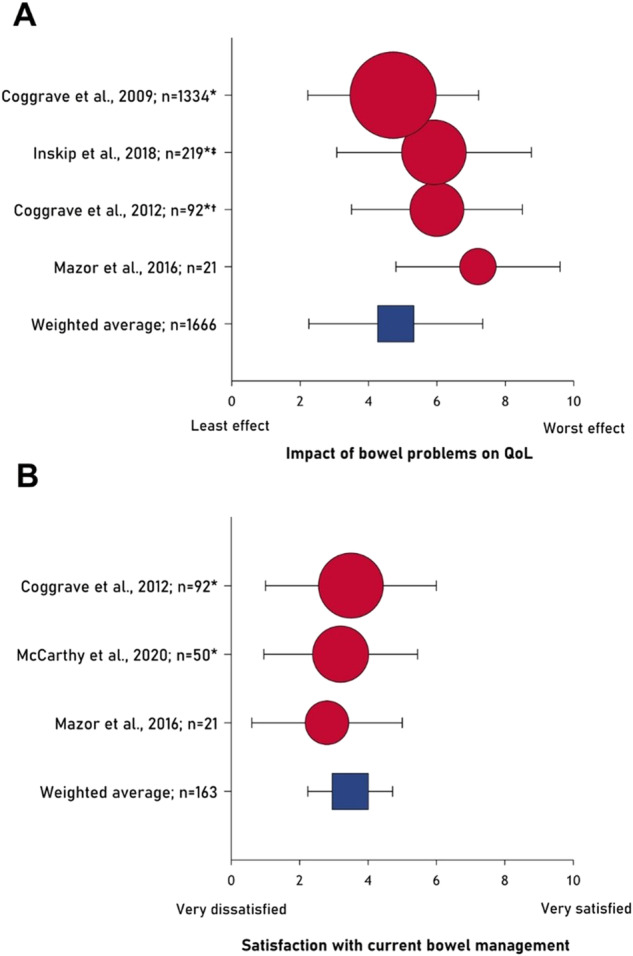
Impact of bowel dysfunction on quality of life. Numerical rating scales evaluating (**A**) the impact of bowel problems on QoL and (**B**) satisfaction with current bowel management. Data are shown as mean ± standard deviation on a 0–10 scale. Bubble size indicates sample size (log transformed). * Denotes mean and standard deviation calculated from median and range; ${{\dagger}}$ indicates the scale direction has been reversed for compatibility; ${{\ddagger}}$ indicates the scale range was from 1-10.

Overall, QoL was negatively impacted by bowel dysfunction [3, 35, 38, 39, 41, 43, 45, 57, 60]. Considering the degree of impact, 27–74% ($\bar{x}$ = 55.5% [n = 992]) of participants in four studies reported a moderate to severe deterioration in QoL due to bowel dysfunction, as measured by study-specific Likert-type scales [3, 29, 41, 45]. Four studies used a 10-point scale to evaluate the impact of bowel dysfunction on QoL (10=worst effect) ($\bar{x}$ = 4.98±2.54 [n = 1666] [3, 6, 56, 59] (Fig. 3A).

FI, constipation, and time to complete bowel care were associated with reduced general QoL. Eight studies evaluated the impact of FI on QoL. An adverse effect of FI on QoL was elucidated by direct question [55, 58, 62], and by statistical association (p < 0.05) [39, 41, 45]. Time to complete bowel care was negatively associated with QoL in three studies [3, 41, 45]. Specifically, clinically problematic bowel care routines ( > 60 min) were associated with deteriorations in QoL (p < 0.01) [41, 45]. Concerning constipation, in two of three studies, constipation was significantly (p < 0.05) associated with reduced QoL scores determined using established QoL questionnaires [49, 50, 53].

The effects of B-AD on QoL were studied in three papers by direct question using non-established questionnaires and/or statistical association [3, 30, 45], and in one study using qualitative interview data [19]. One study found that severity of AD symptoms during bowel care was an independent predictor of QoL (p = 0.036) [3]. Other studies reported no difference in QoL [45], or the degree of bowel program satisfaction [30], between those who did and did not identify B-AD as a problem.

### Bowel constraints on daily living

Seven studies examined how bowel management might constrain activities of daily living. In two studies, 60–85% ($\bar{x}$ = 63.6% [n = 1489]) of participants reported that they “fit their lives around their bowel [management]” a little or a lot [3, 6]. Similarly, another study reported that the median of the sample (n = 92) found their bowel care restricted life “a great deal” [59]. These sentiments were echoed in qualitative interview themes that life was controlled by bowel management routines [63], and frustrations with rigid bowel programs and the time to complete bowel care were barriers to activity and spontaneity [21]. Difficult or disturbed bowel evacuation caused some or major restriction in QoL for 25–32% ($\bar{x}$ = 30.6% [n = 521]) of respondents [52, 58]. Further, activities of daily living were restricted by FI in 15–20% ($\bar{x}$ = 18.9% [n = 502]) of respondents [52, 58], and by constipation for 16% of respondents [58].

### Satisfaction with bowel care

Dissatisfaction with bowel care among individuals with SCI was highlighted in seven studies. In three studies, 23–71% ($\bar{x}$ = 46.9% [n = 743]) of participants were dissatisfied or very dissatisfied with their current bowel management routine [3, 30, 62]. Additionally, one study reported that those with bowel problems were less satisfied with life than those without bowel problems (p = 0.018) [37]. Similarly, three studies used a 10-point scale to measure satisfaction with bowel management (10=very satisfied) ($\bar{x}$ = 3.5 ± 2.5 [n = 1405]) [46, 56, 59] (Fig. 3B). Time to complete (p = 0.001) and ineffective emptying (p < 0.001) were sources of dissatisfaction with bowel care [30].

### Health-related QoL themes

While the exact questions varied, several common themes emerged from the 39 studies that addressed Aim 2. These motifs pertain to physical and mental (including subdomains of emotional well-being, and social functioning) health-related QoL.

### Physical health

The impact of bowel dysfunction on physical health was discussed in 13 studies. Those who managed their bowels independently reported significantly higher SF-12 physical health component summary (PCS; comprising the physical function, role physical, bodily pain, and general health domains) scores compared to those who were dependent on others for care (p < 0.05) [64]. FI was associated with reduced satisfaction with physical health in one study [49] while another found no significant impact of FI on physical health [48]. Individuals with constipation also reported less satisfaction with physical health [49].

Qualitative analyses in two studies revealed challenges with the lack of bowel predictability (both in terms of inconsistencies in care routines and concerns about FI) [21, 63], secondary medical complications due to bowel dysfunction (e.g., fissures and rectal bleeding), and pain or discomfort with ineffective bowel care [21]. In an interview study on the effects of blood pressure dysregulation, of which B-AD was included, respondents described the symptoms of AD as significant physical health concerns, with one participant noting that the headache associated with B-AD was “the worst pain [they’ve] ever had” [19].

**Fig. 4:**
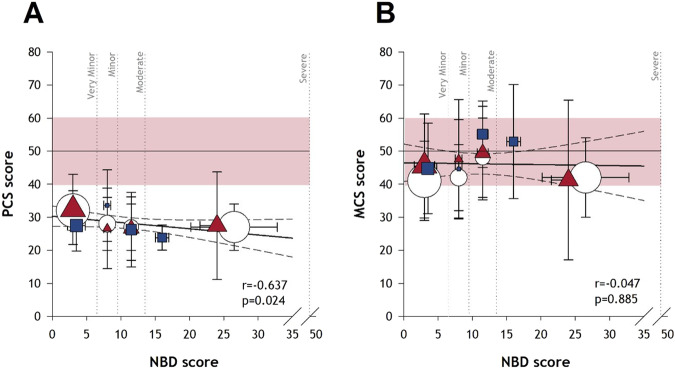
Meta-analysis of Neurogenic Bowel Dysfunction (NBD) score and SF-36 component summary scores. Data for each study are shown as mean ± standard deviation, with the size of the datapoint proportional to the sample size [40, 42, 44]. **A** The physical component score (PCS) was negatively correlated (r = −0.637; p = 0.024) with the NBD score. **B** The mental component score (MCS) was not correlated (r = 0.047; p = 0.885) with the NBD score. The vertical lines denote the range of scores for NBD severity subgroups (very minor, minor, moderate, severe). The horizontal line and red shading denote the mean and standard deviation of American normative SF-36 component summary scores (n = 2474) [32]; a score below this norm indicates reduced QoL. Data from each of the three studies are indicated with different colours and symbols.

Eight studies examined the PCS scores obtained using the SF-36 (n = 6) [34, 36, 40, 42–44] or SF-12 (n = 2) [61, 64]. The presence [34, 36] and severity [40, 42, 44, 61] of bowel dysfunction were significantly (p < 0.05) negatively associated with physical health-related QoL. Four studies reported both the SF-36 PCS and NBD score. A negative linear relationship was found using data from three studies, whereby those with higher NBD scores had more severe impairments as measured by the SF-36 PCS score (r = −0.637, p = 0.024) [40, 42, 44] (Fig. 4A). One study reported no correlation between the SF-36 PCS and NBD scores (r = -0.187, p = 0.235), but raw scores were not reported, thus not included in the meta-analysis [43].

### Mental health

Six studies assessed mental health-related QoL (global mental health, incorporating psychological, emotional and social well-being) using the SF-36, and the presence or severity of bowel dysfunction, with little agreement in results. The mental health component score (MCS; comprising the vitality, social functioning, role emotional, and mental health domains) was negatively associated (p < 0.05) with bowel problems in two studies [36, 43]. One study found only the role emotional domain score was negatively correlated with increasing NBD severity (p = 0.007) [40]. Three studies reported no significant correlations between the MCS and bowel problems [34, 42, 44]. Combining SF-36 MCS and NBD scores from three studies in a meta-analysis revealed no relationship (r = −0.047, p = 0.885) [40, 42, 44] (Fig. 4B). One study found that individuals with bowel problems were less satisfied with their mental health compared to those without bowel problems (p = 0.002) [37]. In another study, satisfaction with psychological health was significantly reduced in those with constipation (p < 0.01), but not in those with FI [49]. Another study found that those without FI were more satisfied (p < 0.05) with their psychological health compared with those who experienced FI monthly or less [48].

#### Emotional well-being

The impact of bowel problems on the emotional function (emotion regulation, awareness, differentiation and expression) of individuals with SCI was addressed in seven studies [21, 30, 46, 57, 62, 63, 65]. In general, emotional well-being was negatively impacted by bowel function [30, 46]. In one study, 66% of respondents felt frustrated, impatient, or restless, 40% felt irritable, and 37% felt tearful or upset sometimes, often, or always due to bowel problems [30]. Qualitative analysis of interview themes reveals that this negative emotional overlay is largely due to the loss of autonomy and privacy in bowel care [21, 63], or the physical experience of bowel care after SCI (e.g., the smells after staining, and the experience of digital stimulation) [21]. An adverse impact of AD on emotional well-being was also reported, whereby AD evoked anxiety and fear; one participant recalled that they could “feel [their] mortality” in an episode of AD [19]. Bowel-related embarrassment was evaluated by five studies. While three studies reported that bowel function [57] and accidents in public [21, 63], caused feelings of humiliation, two studies did not find bowel function to be a strong source of social embarrassment [62, 65].

#### Social function

##### Social engagement

Nine studies addressed the impact of bowel function on social engagement. Bowel management caused moderate to severe interference with the social lives of 39-75% of participants ($\bar{x}$ = 49.4% [n = 2272]) in five studies [3, 6, 45, 47, 52]. Fear of FI was reported as a primary limitation to social participation [21], and increased frequency of FI was associated with reduced social QoL [45]. One study found 68% of those with bowel problems were dissatisfied with their extent of social contact [37]. However, bowel problems were seldom the cause of delay or cancellation of social engagements [30, 62]. Addressing the consequences of B-AD on social function, blood pressure dysregulation was found to be a barrier to community integration [19], and B-AD a hindrance to social activities and activities of daily living [3]. One study found no significant deterioration in social function due to B-AD [45].

#### Relationships

The association between bowel function and personal relationships was discussed in five papers. In two studies, 24–60% ($\bar{x}$ = 28.4% [n = 1795]) of respondents reported bowel function interfered with personal relationships a little or a lot [3, 6]. In interview responses, bowel dysfunction had a clear negative impact on sexual intimacy and relationships. Interviewees expressed a reluctance to pursue new relationships for fear of having to explain or involve their new partner should FI occur [21, 63]. Requiring the assistance of family members during bowel care reportedly strained familial relations [21, 63], and 41% of those with bowel function were dissatisfied with family life overall [37].

#### Lifestyle

Bowel-related limitations to lifestyle and participation were noted in seven studies. Using the FIQL, two studies reported a deterioration in lifestyle due to bowel dysfunction [57, 65]. One study found 25% of respondents avoided attending events with no close accessible bathroom [30]. In two studies, bowel management prevented 42–62% ($\bar{x}$ = 44.9% [n = 1448]) of respondents from staying away from home [3, 6]. Similarly, another study noted that uncertainty of bowel function is a challenge for travel and limits long trips [21]. Two studies reported that bowel problems are a substantial barrier to participation in leisure or sports activities [30, 62].

#### Work

In six studies, bowel dysfunction and management were found to impair the ability to work [3, 6, 21, 30, 62, 63]. The unpredictability of bowel function and FI was a limiting factor for the pursuit of education and employment [21], and was associated with frustrations surrounding loss of independence [63]. Accordingly, bowel problems limited work attendance for 12–26% ($\bar{x}$ = 24.4% [n = 1313]) of respondents [6, 30]. One study found that bowel management was a barrier to working away from home for 41% of participants, and B-AD was a hindrance to work [3]. Despite a reduction in work attendance due to bowel dysfunction, one study highlighted that those who worked were generally satisfied with their quality of work and their bowel management did not limit their standard of work [30].

## Discussion

This paper provides the first systematic review using quantitative and qualitative evidence to examine the full landscape of bowel care challenges post-SCI and their impact on QoL. Our analyses emphasise the high prevalence of bowel dysfunction, FI, constipation, and B-AD after SCI. Times to complete bowel care are often problematically long. The negative impacts of these bowel care challenges on QoL are wide-ranging and significant, leading to bowel care dissatisfaction, life restriction, and impairing physical, emotional, and social health-related domains of QoL [3, 33, 39, 47].

### FI and fear of accidents is common and negatively impacts QoL after SCI

FI or the risk of FI was a consistent QoL-reducing problem for over half the participants studied [4, 21, 39, 63], revealing mistrust in the ability of bowel routines to prevent accidents. Despite the fear of FI, prevention and management tools such as incontinence pads and anal plugs are not used regularly by most individuals with SCI [3]. This may reflect stigma surrounding the use of incontinence pads, or concerns that anal plugs may trigger a reflexive bowel movement. Fear of worsening incontinence is a barrier to changing bowel care practices, perpetuating a cycle of apprehension to change and inaction [66]. The restrictive impact of the fear of FI is most evident in social function-related QoL. FI has been noted as a barrier for the pursuit of work (travel, time flexibility, and bathroom accessibility) [21, 66] and a reason participants hesitate to start new relationships [21, 63]. FI can also complicate perianal wound care and further reduce QoL [45]. Women with SCI report concerns that penetrative sex may trigger involuntary bowel movement [41, 63, 67], highlighting the need to consider the interplay between autonomic dysfunctions after SCI to improve QoL.

### Constipation is prevalent with multifaceted effects on QoL

Despite occurring with a similar prevalence to FI, constipation did not provoke a uniformly negative impact on QoL. This may reflect that individuals with SCI do not sense the discomfort of impacted stool if the sensory afferents from the stomach, small intestine, and colon arise below the level of injury [68]. However, constipation may be an intermediary to other factors that reduce QoL; the distended bowel may trigger AD, constipation may increase the bowel care duration if a more extensive bowel routine is required (e.g., laxatives, stool softener, manual evacuation), and risk of FI rises if constipation results in irregular and unsuccessful bowel care [66]. Additionally, constipation is likely exacerbated by other behavioural changes including fluid restriction, a strategy employed by some individuals with SCI for the management of urinary incontinence [66]. This reflects the interconnectivity between bladder and bowel care and highlights the challenges with management of the widespread and interrelated autonomic problems after SCI, which may have additive detrimental impacts on QoL.

### Symptomatic B-AD negatively impacts QoL in those with high-level injuries

Evidence for the impact of B-AD on QoL was contradictory, likely due to the range of lived experiences of AD, from symptomatic and distressing, to asymptomatic or ‘silent’. For symptomatic individuals, B-AD was an independent determinant of QoL [3] and is among the leading reasons why some individuals elect to manage bowel care with an ostomy [30, 59, 69]. B-AD may be particularly problematic because the long duration of care means the stimulus for AD is prolonged and the overall AD burden is high [70]. Asymptomatic B-AD is unlikely to impact QoL, however, even with silent AD the risk of cardiovascular morbidity and mortality remains [13–15]. The variable presentations of B-AD highlight the importance of reducing B-AD as a clinical target for all at-risk individuals [70]. Periodic blood pressure monitoring could provide a window into potential blood pressure spikes during bowel care and may aid clinical decisions around optimal care routines. Bowel care management should be personalised and optimized, with consideration of resource availability and financial impacts of the care routines adopted [71].

### Prolonged care routines impair QoL after SCI

Time to complete bowel care was an important predictor of QoL and was intertwined with other bowel dysfunction problems. Prolonged bowel care can be the consequence of constipation or an increased number of bowel management approaches, and long bowel care itself may induce a greater burden of AD [70] and an increased risk for pressure wounds [72]. Time to complete is an essential metric of the success of bowel care but is only considered to be clinically problematic when the routine exceeds 1 h [20]. However, over 40% of participants required >30 minutes to complete bowel care [3, 38, 45, 46, 52, 59], far longer than typical care routines for the general population, and this was considered unacceptably long by those living with SCI [21]. These data affirm that the time spent doing bowel care is time not spent doing something else, and highlight the negative impact of time to complete care on QoL and activities of daily living [73]. These data highlight the importance of discussing bowel care options with people with shorter ( < 1 h) care routines that do not meet clinical guidelines for problematic care, but still negatively and meaningfully impact QoL. Certainly, time to complete is a simple and accessible marker for caregivers, clinicians, and people living with SCI to understand if bowel care routines are effective and appropriate [3, 30, 41]. Increases in time to complete should be considered a sign of problematic care.

### Bowel dysfunction has a profound effect on multiple domains of QoL

Our results suggest that bowel dysfunction has a particularly negative impact on physical health. This may reflect that the primary scope of the instruments used evaluated the severity of bowel dysfunction from a physical health standpoint. Nevertheless, ineffective bowel care is a key concern for physical health-related QoL, and problems with constipation, FI, and B-AD should be prioritised in clinical care. Importantly, the ability to independently perform care routines was a critical factor for physical health-related QoL [51, 63, 64], and has been emphasised in clinical guidelines [20]. Independence of care allows bowel care to remain a private undertaking, improving social and emotional well-being and limiting reliance on others to complete bowel care [63]. As autonomy of care depends on mobility and hand function [50], bowel care routines should be individually crafted to prioritise and maximise independence.

Associations between bowel dysfunction and mental health were mixed. This may reflect that, with time, individuals with SCI adjust to the expectation that bowel care will be among the challenges they face [66]. However, this does not negate the general dissatisfaction with bowel routines and the associated mental health burden that was a pervasive theme in the studies identified. Common concerns were the negative impact of bowel dysfunction on emotional well-being (largely reflecting loss of autonomy and privacy in conducting care), and the adverse effect of anxiety and fear related to episodes of B-AD and FI. Bowel dysfunction also adversely affected social functioning, hindering community engagement, social participation, work attendance, intimacy, and relationships. Qualitative [21, 63], but not quantitative [62, 65], analyses highlighted embarrassment and humiliation as concerns in the context of bowel management, highlighting the importance of mixed methodological approaches when considering bowel management.

Individuals with SCI experience a hierarchy of care, whereby health goals are prioritised, often necessarily based on the most clinically urgent need [66]. This may mean that, despite the significant QoL consequences, sub-optimal but clinically acceptable bowel routines receive little attention until critically dysfunctional. This may be compounded by the reality that the reasons for adopting a particular care routine may focus on financial constraints, healthcare provider preference, and care aide availability, among other factors, rather than the preference of the individual. These data confirm that improving satisfaction with bowel care is a priority for those living with SCI [7], and access to resources and supports for bowel care should be prioritised. Understanding the limitations of appointment times with health care providers, peer support can be useful for discussions of bowel care routines, providing a trustworthy alternative route for knowledge dissemination and empowerment for individuals with SCI to improve their bowel care [66, 74, 75].

### Methodological considerations

We conducted a comprehensive review examining the relationships between bowel care and QoL after SCI, compiling results from studies with diverse methodologies. One key strength of our approach is the inclusive synthesis of quantitative and narrative evidence. Recognising the importance of patient-oriented research, we integrated narrative evidence through the use of secondary analysis of qualitative data using a systematic approach to ensure the lived experiences and perspectives of individuals living with SCI were appropriately reflected in the data [33].

Another strength is the transparent reporting of all studies, with full data extraction provided in the supplementary files, enabling the reader to self-evaluate the evidence upon which our conclusions were made. In addition, we employed a quality evaluation instrument that is particularly suited to our mixed methods approach [27], which incorporated data from numerous instruments and considered quantitative and qualitative evidence. While we note the strengths of this tool, one drawback to a general tool that permits appraisal of studies with broad methodology is that it does not permit detailed evaluation of specific components or facilitate critique of specific data visualisation or reporting (which is highly variable in studies with diverse methodologies). In addition, this tool evaluates the quality of the study with respect to the original study aims, not placed in the context of the present meta-analysis. Nevertheless, in general the tool provided an effective means to appraise article quality, which was high across multiple domains of evaluation. Areas for quality improvement were identified in the involvement of stakeholders, advisory groups, or people with lived experience in informing the work, a key component of integrated knowledge translation [76], as well as in explicit discussion of strengths and limitations to the work. Reduced scores in this area predominantly reflected a focus on limitations to the work, without clearly highlighting research strengths, or focus only on the obvious impact of a small sample size on statistical power, without considering the broader aspects of the study in a more fulsome manner.

We could not stratify results based on SCI or participant characteristics (e.g., sex, age, level of injury, ASIA classification, duration of injury) as these data were either not reported or reported inconsistently. Where available we provided these stratifications in the data tables for the interested reader. Despite reporting challenges, there were suggestions that individuals with higher and more complete lesions experience more severe bowel dysfunction, although those with lower level injuries were predisposed to FI [3, 40, 42, 47, 55]. There were also some reports that women [6, 52], those with longer durations of injury [55], and older individuals [55, 58] experienced more severe bowel dysfunction with a greater impact on QoL, affirming the adverse impact of aging with SCI and age-related comorbidities on the secondary complications of SCI [77–79].

Based on known pathophysiology, only those with high-level injuries are at risk for B-AD and yet in some studies B-AD data were reported for all individuals [30, 38, 45, 46, 52], including those not at risk for B-AD based on lesion level. This may have led to underestimation of the true prevalence and severity of B-AD in those for whom it is a pertinent consideration.

There is little agreement on the best method for bowel emptying. In this review, data were not stratified by bowel care technique, and we did not aim to evaluate the effectiveness of care methodology but rather care impacts on QoL.

We were not able to complete an extensive meta-analysis due to the lack of common outcome measures, highlighting the need for future research to employ commonly used and established measurement tools to facilitate data aggregation and comparison between studies. When considering B-AD, while there is a standard definition for AD [80–82], there is opportunity in the literature to explicitly state the operational definition, assessment and quantification of AD and symptoms of AD used in each study.

## Conclusions

Bowel dysfunction and bowel care challenges are prevalent and disabling for individuals with SCI, with a profoundly negative impact on QoL. Bowel dysfunction has detrimental impacts on physical health, emotional wellbeing, and social function that result in lower satisfaction with bowel routines. Priorities for bowel care routines should be to shorten time to complete, maximise independence, and reduce the risk of FI, constipation, and B-AD to improve QoL for those living with SCI.

### Supplementary information

Supplemental File

## Data Availability

All data were extracted from previously published works and are provided for the interested reader in the article figures and tables.
