# Supplementary material for: Bowel burdens: a systematic review and meta-analysis examining the relationships between bowel dysfunction and quality of life after spinal cord injury
Source: Spinal Cord. 2024 Jul 16;62(9):495–506. doi: 10.1038/s41393-024-01002-8 (PMC11368817; doi:10.1038/s41393-024-01002-8)
Supplement: Supplementary file 1 — Supplemental File [file 41393_2024_1002_MOESM1_ESM.docx]

**Supplemental Table 1** *Database search strategy.* *Denotes a wildcard truncation symbol used to search all variations on spelling.

| **Database** | **Search Terms** | **# of Results** |
| --- | --- | --- |
| PubMed | (spinal cord injuries[MeSH Terms] OR spinal cord ischaemia[MeSH Terms] OR “sci” OR “spinal cord inj*” OR central cord injury syndrome[MeSH Terms] OR spinal cord diseases[MeSH Terms]) AND (“quality of life” OR “QoL” OR “morbidity” OR “complications” OR “health related quality of life” OR “HRQoL” OR “participation” OR “activities of daily living” OR “SF-36” OR “HRQOL-14” OR “WHOQOL-BREF” OR “social participation” OR “community participation” OR “life satisfaction” OR “mental health” OR “wellbeing” OR quality of life[MeSH Terms] OR patient satisfaction[MeSH Terms] OR community participation[MeSH Terms] OR social participation[MeSH Terms] OR emotions[MeSH Terms] OR morbidity[MeSH Terms] OR activities of daily living[MeSH Terms] OR mental health[MeSH Terms]) AND (neurogenic bowel[MeSH Terms] OR fecal incontinence[MeSH Terms] OR autonomic dysreflexia[MeSH Terms]) | 151 |
| Web of Science | (spinal cord injuries OR spinal cord ischaemia OR “sci” OR “spinal cord inj*” OR central cord injury syndrome OR spinal cord diseases) AND (“quality of life” OR “QoL” OR “morbidity” OR “complications” OR “health related quality of life” OR “HRQoL” OR “participation” OR “activities of daily living” OR “SF-36” OR “HRQOL-14” OR “WHOQOL-BREF” OR “social participation” OR “community participation” OR “life satisfaction” OR “mental health” OR “wellbeing” OR patient satisfaction OR emotions OR mental health) AND (neurogenic bowel OR fecal incontinence OR autonomic dysreflexia OR autonomic hyperreflexia) | 498 |
| PsycInfo | (see search strategy for Web of Science) | 40 |
| CINAHL | (see search strategy for Web of Science) | 576 |
| Embase | ('spinal cord injuries' OR 'spinal cord ischaemia' OR sci OR 'spinal cord inj*' OR 'central cord injury syndrome' OR 'spinal cord diseases') AND ('quality of life' OR QoL OR morbidity OR complications OR 'health related quality of life' OR HRQoL OR participation OR 'activities of daily living' OR 'SF 36' OR ‘HRQoL 14' OR 'WHOQOL BREF’ OR 'social participation' OR 'community participation' OR 'life satisfaction' OR wellbeing OR 'patient satisfaction' OR emotions OR 'mental health') AND ('neurogenic bowel' OR 'fecal incontinence' OR 'autonomic dysreflexia' OR 'autonomic hyperreflexia') | 777 |

**Supplemental Table 2** *Table of instruments used.* Abbreviations: HRQoL, health-related quality of life; QoL, quality of life.

| **(A) Instrument**  **(B) Instrument type**  **(C) Generic or condition specific**  **(D) Frequency (# studies)** | **Assessment details and domains** | | **Scoring system** | |  |
| --- | --- | --- | --- | --- | --- |
| (A) **Autonomic Dysfunction Following Spinal Cord Injury (ADFSCI) (modified)**  (B) Autonomic dysfunction-specific  (C) Condition specific  (D) 1 [Stoffel et al., 2021] | The ADFSCI is a 24-item questionnaire divided into 4 sections: demographics, medication, autonomic dysreflexia (AD), and hypotension (1).  Note that Stoffel et al., 2021, utilised a subsection of the AD-related questions in the ADFSCI instrument. These included domains of bowel function, bladder function, sexual activity, exercise, and unprovoked activities (2). | | The AD and hypotension domains score the frequency and severity of hyper- or hypotensive symptoms under different circumstances using a 5-point scale, ranging from 0 (least frequent and severe symptoms) to 4 (most frequent and severe symptoms) (1).  In Stoffel et al., 2021, item scores were summed to generate a total AD score from 0 (least severe) to 24 (most severe) (2) . | | |
| (A) **Bowel Continence Score (BCS)**  (B) Bowel care-specific  (C) Generic  (D) 1 [Kannisto & Rintala, 1995] | | The BCS consists of 7 items assessing the frequency of defecation, rectal sensation, soiling, ability to hold back defecation, faecal consistency, discrimination between stool texture, and need for therapy (3). | | Each item is scored from 0-2 with higher scores indicating more typical bowel habits. The sum of item scores produces a total score ranged 0-14, with scores binned into levels of continence control: Poor, 0-4; Fair (marked limitations in social life), 5-9; Good (social continence), 10-13; normal bowel habits, 14 (3). | |
| (A) **Burwood Quality of Life (Burwood QoL) Survey (modified)**  (B) QoL, bowel care-specific  (C) Generic  (D) 1 [Pardee et al., 2012] | | In the modified Burwood QoL survey, 18 items covered 4 domains: systemic symptoms (4 items), emotional function (7 items), social function (4 items), and work function (5 items). The subscales of the Burwood QoL questionnaire were restructured in the study by Pardee et al. (2012), although the wording and response categories were retained. The modified version maintained high reliability during pilot testing (a=0.82) (4). | | Items are rated on a scale of 1 ”never” to 5 ”always” (range 18-90). Lower scores indicate better QoL (5). | |
| (A) **Craig Handicap Assessment and Reporting Technique (CHART)**  (B) HRQoL  (C) Generic  (D) 1 [Hwang et al., 2017] | The CHART questionnaire assesses 6 domains using 32 items: physical independence, cognitive independence, mobility, occupation, social integration, and economic self-sufficiency (6). | | Each domain is scored from 0-100, and the total CHART score is summed to range from 0-600. Higher scores indicate a lesser degree of disability, or greater degree of social participation (6). | |  |
| (A) **EQ-5D-3L**  (B) HR-QoL  (C) Generic  (D) 1 [Van Doorn et al., 2022] | | The EQ-5D-3L is a generic measure of health status. It describes the health state of an individual across 5 dimensions: mobility, self-care, usual activities, pain/discomfort, and anxiety/depression. The EQ-5D-3L also includes a single-item visual analogue scale (EQ VAS) (7). | | Each EQ-5D-3L dimension has three levels of severity, indicating (1) no problems, (2) some problems, and (3) extreme problems. Responses to the five dimensions can be summarized as a 5-digit health state (e.g., 12112) and converted into a single summary score (a health utility value) interpreted on a scale with anchors at 0 (dead) and 1 (full health(7) Negative values represent health states worse than dead. The EQ VAS asks participants to rate their health on a scale from 0 (“The worst health you can imagine”) to 100 (“The best health you can imagine”)(7). | |
| (A) **Fecal Incontinence QoL (FIQL)**  (B) HRQoL, bowel specific  (C) Generic  (D) 2 [Smith & Decter, 2015; Van Doorn et al., 2022] | The FIQL scale assesses the impact of FI. It consists of 29 items across 4 domains: lifestyle (10 items), coping/behavior (9 items), depression/ self-perception (7 items), embarrassment (3 items) (8). | | Most items are assessed using 4 response categories that correspond with a 1-4 rating system (ie most (1) to none (4) of the time; strongly agree (1) to strongly disagree (4)). Responses are averaged for each domain. There is no established method for calculating an overall score. Higher scores indicate a more favorable QoL (8). | |  |
| (A) **Fecal Incontinence Severity Index (FISI)**  (B) Bowel care-specific  (C) Generic  (D) 1 [Mazor et al., 2016] | The FISI assesses the severity of fecal incontinence as a matrix across four incontinence types: “Gas”, “Mucus”, “Liquid” or “Solid” (9). | | Incontinence types are evaluated by how often they occur, ranging from “Never” to “2 or More Times a Day”. The outcome is a score from 1 “least severe condition” to 20 “most severe condition” (9) . | |  |
| (A) **Functional Independence Measure (FIM), Sphincter Control subdomain**  (B) Bowel care-specific  (C) Generic  (D) 2 [Leduc et al., 2002; Ture et al., 2022] | The FIM score consists of 18 items in two subscales. The motor subscale includes sub-domains of self-care, sphincter control, transfers, and locomotion. The cognition subscale includes domains for communication and social cognition (10).  Leduc et al. (2002) utilised the bowel item of the sphincter control sub-domain as a measure of independence in bowel management (11). | | Individual items are scored from 1 “total assistance” to 7 “complete independence”. Subdomain scores are determined by the sum of its constituent item scores, and a total FIM score by the sum of all item scores. The sphincter control sub-domain score ranges from 1-7 with higher scores indicating greater independence in bowel management (10). | |  |
| (A) **Health Utilities Index Mark III (HUI3)**  (B) HRQoL  (C) Generic  (D) 1 [Craven et al., 2012] | The HUI3 is a generic measure of health status. The classification system comprises eight attributes: vision, hearing, speech, ambulation, dexterity, emotion, cognition, and pain (12). | | All health states defined by the HUI3 classification system are associated with a health utility value. The value represents the relative value society places on living in the health state. Health utility values are interpreted on a scale from 0 (dead) to 1 (full health). Negative values represent health states worse than dead (12). | |  |
| (A) **Inflammatory Bowel Disease Questionnaire (IBDQ) (modified)**  (B) HRQoL, bowel care-specific  (C) Generic  (D) 1 [Luther et al., 2005] | The IBDQ measures HRQoL in 32 items forming 4 domains: bowel symptoms (10 items), systemic symptoms (5 items), emotional function (12 items), social function (5 items) (13).  Note that in Luther et al., 2005, the wording in 7 items was minimally modified to better suit the NBD-related issues of individuals with SCI (14). | | Individual items are scored from 1 "worst" to 7 "best" situation and summed for a total score (range 32-244). Higher scores indicate a more favorable QoL (13). | | |
| (A) **International Classification of Functioning, Disability, and Health (ICF) framework**  (B) HRQoL  (C) Generic  (D) 3 [Burns et al., 2015; Carlozzi et al., 2013; Nevedal et al., 2017] | Developed by the World Health Organization, the ICF framework provides standardised structure and language for describing functioning and disability. The framework organises themes into five concepts that can act as barriers or facilitators of function: body functions and structures, activities, participation, environmental factors, and personal factors. This framework provided rigor and structure to interpret narrative responses (15). | | Not applicable. | |  |
| (A) **International SCI Bowel Basic Data Set (ISCIBDS-bowel) (modified)**  (B) HRQoL  (C) Condition specific  (D) 1 [Inskip et al., 2018] | | The ISCIBDS-bowel questionnaire was designed by a working group including members from the American Spinal Injury Association and the International Spinal Cord Society. It uses 12 items in a standardised format to collect and report bowel function information (16)  Note that in Inskip et al., 2018, questions regarding cardiovascular signs and symptoms, nutrition, hydration, medication use, and participant demographics were added to the questionnaire (17). | | Not applicable. | |
| (A) **International SCI QoL Basic Data Set (ISCIBDS-QoL)**  (B) HRQoL  (C) Condition specific  (D) 2 [Elmelund et al., 2019; Adriaansen et al., 2016] | The ISCIBDS-QoL questionnaire involves 3 numeric self-ratings to assess satisfaction with general QoL, physical health, and psychological health in the past 4 weeks (18). | | Each item is scored from 0 "completely dissatisfied" to 10 "completely satisfied". A total score a can be produced by averaging the 3 item scores (range 0-30) with higher scores denoting greater satisfaction (18). | |  |
| (A) **Knowles, Eccersley, Scott Symptom Score (KESS)**  (B) Bowel care-specific  (C) Generic  (D) 1 [Mazor et al., 2016] | The KESS is an 11-question symptom scoring questionnaire that scores symptoms of constipation across 11 areas (“Duration of constipation”, “Laxative use”, “Frequency of bowel movement”, “Unsuccessful evacuatory attempts”, “Feeling incomplete evacuation”, “Abdominal pain”, “Bloating”, “Enemas/Digitation”, “Time taken”, “Difficulty evacuating”, and “Stool consistency”) (19) | | Scores range from 0 “symptom free state” to 39 “most symptomatic” (19). | |  |
| (A) **Neurogenic Bowel Dysfunction score (NBD)**  (B) Bowel care-specific  (C) Generic  (D) 7 [Erdem et al., 2017; Gong et al, 2021; Koyuncu et al., 2017; Krogh et al., 2006; Liu et al., 2009; Pires et al.,2012; McCarthy et al., 2020] | The NBD score measures bowel function in 10 items assessing frequency and duration of bowel care, use of bowel management techniques (digital stimulation, medication), and issues with bowel care (constipation, incontinence, flatus incontinence, perianal skin problems, symptoms compatible with B-AD) (20). | | The scores of each item are weighted unevenly, but produce a total score from 0-47. Higher scores indicate a greater degree of dysfunction. NBD scores are separated into categories of NBD severity: Very minor, score 0-6; Minor, 4-9; Moderate, 10-13; Severe, 14-47 (20). | |  |
| (A) **Physician Global Assessment (PGA)**  (B) HRQoL  (C) Generic  (D) 1 [Erdem et al., 2017] | The PGA provides a measure of the patient's own assessment of the impact of their condition. It is a singular question, in this case recorded on a 10cm visual analog scale of subjective disease impact (21). | | The PGA is recorded on a 10cm visual analog scale (range 0-10) (21). | |  |
| (A) **Satisfaction with Life Questionnaire (LiSAT-11)**  (B) QoL  (C) Generic  (D) 3 [Locke et al., 2019; Sweet et al., 2014; Jorgensen et al., 2021] | The LiSAT-11 is used as a measure of life satisfaction and consists of 11 questions with 1 question per domain. The domains assessed are satisfaction with physical health, psychological health, self-care, vocation, finance, leisure, sex, partner relations, family life, contact with friends, and life as a whole (22). | | Each item is scored on a 6 point scale from 1 "very dissatisfied" to 6 "very satisfied". A total score a can be produced by averaging the item scores. (range 11-66). Higher scores indicate greater life satisfaction (22). | |  |
| (A) **Spinal Cord Injury Quality of Life (SCI-QoL) Bowel management difficulties item bank**  (B) QoL, bowel care-specific  (C) Condition-specific  (D) 2 [Gong et al., 2021; Gorman et al., 2021] | The bowel management subscale of the SCI-QoL questionnaire consists of 12 items (23). | | Each item is rated on a 5-point Likert scale from 1 "never" to 5 "always". A total score is produced from the sum of the item scores (range 12-60). Higher scores indicate poorer excretion-related QoL (23). . | |  |
| (A) **12-item Short Form health survey (SF-12)**  (B) HRQoL  (C) Generic  (D) 3 [Hicken et al., 2001; Locke et al., 2019; Stoffel et al., 2021] | The SF-12 is an abbreviated version of the 36-item Short Form health survey (SF-36) consisting of 12 items. It assesses 8 domains: physical functioning (PF), role limitations due to physical health (RP), bodily pain (BP), general health (GH), vitality (VT), social functioning (SF), role limitations due to emotional health (RE), and mental health (MH). Physical and mental health component summary scores (PCS, MCS) can be generated from the domain scores (24,25). | | Domain scores are converted into a 0-100 scale, where higher scores indicate better functioning. Norms-based algorithms are used to generate the PCS and MCS scores. The PCS is composed of the domain scores of PF, RP, BP, and GH. The MCS is composed of the domain scores of VT, SF, RE, and MH (24,25). | |  |
| (A) **12-item Short Form health survey version 2 (SF-12v2)**  (B) HRQoL  (C) Generic  (D) 1 [Hwang et al., 2017] | The SF-12v2 is updated version of the SF-12, with amendments to wording and response options (26). | | Domain scores are converted into a 0-100 scale, where higher scores indicate better functioning. Norms-based algorithms are used to generate the PCS and MCS scores (26). These component summary scores remain comparable to those generated from the SF-12. | | |
| (A) **36-item Short Form health survey (SF-36)**  (B) HRQoL  (C) Generic  (D) 7 [Lombardi et al., 2010; Noonan et al., 2008; Koyuncu et al., 2017; Westgren & Levi, 1998; Liu et al., 2009; Erdem et al., 2017; Ture et al., 2022] | The SF-36 contains 36 items across 8 domains: PF (10 items), RP (4 items), BP (2 items), GH (5 items), VT (4 items), SF (2 items), RE (3 items), and MH (5 items). From these 8 domains, physical and mental health component summary scores (PCS, MCS) can be generated (24,27). | | Domain scores are transformed into a 0-100 scale, with each item carrying equal weight. Higher scores indicate better functioning and/or lesser limitation. The summary component scores are generated using the aggregated scores of 4 domains and normalized to the US population mean (standard deviation): 50. The PCS is composed of the domain scores of PF, RP, BP, and GH. The MCS is composed of the domain scores of VT, SF, RE, and MH (24,27). . | |  |
| (A) **Wexner Score**  (B) Bowel care-specific  (C) Generic  (D) 2 [Lombardi et al., 2010; Lynch et al., 2000] | The Wexner Score evaluates the severity of incontinence. Its 5 items assess the type of incontinence (solid, liquid, gas), management technique, and lifestyle alteration (28). | | Each item is scored by the frequency experienced, from 0 “Never” to 4 “Always” to produce a total score ranging from 0 “perfect continence” to 20 “complete incontinence” (28). | |  |

**Supplemental Table 3** *Impact of SCI on bowel function.* Where appropriate, data are reported as mean ± standard deviation, or proportion of respondents (%) identifying with the category. * denotes data calculated based on metrics provided. Sample sizes are provided for the measure as a whole, and for specific measures as applicable in cases where not all participants completed every metric.

| **Study / Instrument / [n]** | **Bowel dysfunction** | **Bowel care frequency /**  **Time to complete** | **Incontinence / Constipation** | **B-AD** | **Additional Results** |
| --- | --- | --- | --- | --- | --- |
| **Adriaansen et al., 2016**  Questionnaire: non-established  [n=282] |  |  | **Incontinence:** 16%  **Constipation:** 25% |  |  |
| **Carlozzi et al., 2013**  Interviews, ICF framework (modified): non-established  [n=27] |  |  |  | Conceptual model finds a full bowel or bladder to be a cause of AD, leading to symptoms of fatigue, pounding headaches, vision problems, and feeling faint. |  |
| **Craven et al., 2012**  SCI secondary conditions scale (modified): non-established  [n=357] | Self-report bowel dysfunction 42% |  |  |  | Most (78%) [n=23] individuals had problems with at least two of bladder, bowel, or sexual dysfunction. |
| **Coggrave et al., 2012**  Questionnaire: non-established  [n=92] |  | **Time [n=90]:** ≤15 min 5%; 16-30 min 11%; 31-60 min 31%; >60min 46% |  |  | Participants reported the following reasons for choosing stoma formation: 68% prolonged bowel care, 53% FI, 29% constipation, 21% B-AD. |
| **Coggrave et al., 2009**  Questionnaire: non-established  [n=1334] | Most (99%) of respondents reported experiencing at least one of the following problems with their bowel: constipation, FI, AD, haemorrhoids, abdominal distension, abdominal pain, prolonged bowel care. | **Time:** >60 min 14% | **Incontinence:**  >Monthly 8%  **Constipation:** 39% | Of those at risk of AD (injury above T7), 10% often/ always and 36% occasionally experience B-AD. | Those who reported more bowel problems also tended to report a longer duration of bowel care (p<0.001).  Women reported more constipation (p<0.001), abdominal distension (p<0.001), abdominal pain (p=0.001), and more bowel problems in total (p=0.002). |
| **Elmelund et al., 2019**  Questionnaire: non-established  [n=684] |  | **Time [n=585]:** ≤5 min 36%; 6-30 min 54%; >30 min 10% | **Incontinence [n=599]:** 35%  Never 65%; <Monthly 14%, Monthly 9%; Weekly 8%, Daily 4% |  |  |
| **Erdem et al., 2017**  NBD score  [n=42] | NBD Score: 15.6±2.5*  NBD Severity: 38% very minor; 33% minor; 10% moderate; 19% severe |  |  |  |  |
| **Gong et al., 2021**  NBD score  [n=101] | NBD Score: Not reported.  NBD Severity [n=64]: 36% very minor; 13% minor; 19% moderate; 33% severe |  | **Incontinence:** 53% |  |  |
| **Gorman et al., 2021**  Questionnaire: non-established [n=49] |  | **Time:** 5-60 min 80%; 61-180 min 18%  Accumulated bowel care time needed in the past week: 1-6 hrs 80%; 6-8 hrs 16% |  |  | Enema, oral medication and/or manual digital stimulation needed for each bowel evacuation in the past week: never to a few times for 57%, most to every time for 41%. |
| **Hwang et al., 2017**  Questionnaire: non-established  [n=131] |  |  | **Incontinence:** 18% |  |  |
| **Inskip et al., 2018**  ISCIBDS – bowel (modified): non-established  [n=231] |  | **Frequency:** Daily 51%; 1-6 per week 48%; > once per week 1%  **Time:** ≤30 min 57%; 31-60 min 18%; >60 min 24% | **Incontinence [n=219]:** 78%*  Never 12%; < Monthly 45%; Monthly 17%; Weekly 11%; Daily 5% | Of those at risk of AD (injury above T7), 74% reported at least one symptom of AD during their bowel care routine. | Frequency of fecal incontinence was significantly higher in those with lower motor neuron NBD than with higher motor neuron NBD (p=0.0033).  Predictors of severity of AD symptoms during routine include the number of bowel management approaches used (p<0.001) and fatigue (p<0.001). |
| **Jorgensen et al., 2017**  Questionnaire: non-established  [n=123] |  |  | **Incontinence:** 12%  **Constipation:** 11% |  | |
| **Jorgensen et al., 2021**  Questionnaire: non-established  [n=78] | Self-report bowel dysfunction 47% |  |  |  |  |
| **Kannisto & Rintala., 1995**  BCS  [n=35] |  | **Frequency:** 2-3 per week 77% | **Incontinence:** 72%*  Never 29%; Sometimes 66%; Daily 6%  BCS 8±2.65  **Constipation:** Prolonged colonic transit time 54% |  | Those with complete low lesions (T7-S5) had higher BCS scores, indicating greater continence control than those with complete high lesions (p>0.05). |
| **Kim et al., 2012**  Questionnaire: non-established  [n=388] |  | **Frequency [n=383]:** Daily 22%; Every other day 26%; 1-3 per week 40%; < once per week 13%  **Time [n=383]:** ≤15 min 37%; 16-30 min 28%; 31-60 min 26%; >60 min 9% | **Incontinence [n=379]:** 67%*  < Monthly 41%; Monthly 13%; Weekly 6%; Daily 7% | Symptoms compatible with B-AD (generalized discomfort during defecation) were reported by 42% of respondents. |  |
| **Koyuncu et al., 2017**  NBD score  [n=55 | NBD Score: 9.1±5.1  NBD Severity: 35% very minor; 11% minor; 29% moderate; 26% severe |  |  |  | Individuals with complete lesions (AIS A) reported higher NBD scores than those with incomplete lesions (AIS B,C,D) (10.8±4.3 vs. 7.8±5.3; p=0.044).  NBD scores did not significantly differ with lesion level (p=0.08) or sex (p=0.524). |
| **Krogh et al., 2006**  NBD score  [n=424] | NBD Score: 12.8±5.2*  NBD Severity: 28% very minor; 15% minor; 28% moderate; 29% severe |  |  |  |  |
| **Krogh et al., 1997**  Questionnaire: non-established  [n=424] |  | **Time [n=413]:** ≤15 min 52%; 16-30 min 24%; 31-60 min 16%; >60 min 9% | **Incontinence [n=416]:** 75%  < Monthly 56%; Monthly 14%; Weekly 4%; Daily 1% | Symptoms compatible with B-AD (physical uneasiness, headache, or perspiration before and during defecation) were reported by 25 % of respondents. |  |
| **Leduc et al., 2002**  FIM Sphincter control subdomain  [n=30] | FIM sphincter control subdomain:  score=6 63%;  score>6 37% | **Time:** <60 min 90%; ≥60 min 10% | **Incontinence:** 57%  **Constipation:** colonic transit time 2.7 days; Prolonged transit time 47% |  |  |
| **Liu et al., 2009**  NBD score  [n=128] | NBD Score: Not reported.  NBD Severity [n=123]: 35% very minor; 18% minor; 14% moderate; 33% severe |  |  |  |  |
| **Locke et al., 2019**  Questionnaire: non-established  [n=1529] |  |  | **Incontinence:** 63%*  Never 36%; <Monthly 43%; Monthly 11%; Weekly 5%; Daily 4%; Don't know 1%  **Constipation:** 81%*  Never 18%; <Monthly 30%; Monthly 21%; Weekly 16%; Daily 14%; Don't know 1% |  |  |
| **Lombardi et al., 2010**  Wexner Score  [n=28] |  |  | **Incontinence:** Wexner score 16.7±3*  Chronic FI: 13.09  Chronic constipation: 19.91  **Constipation:** Prolonged transit time 48%  Global intestinal transit time 4.5 days;  Chronic FI group: 3.15 days  Chronic constipation group: 4.5 days |  |  |
| **Lynch et al., 2000**  Wexner Score  [n=467] |  | **Frequency:** Mean 7 bowel movements/week  **Time:** ≥15 min 61% | **Incontinence:**  Daily 8%  Complete SCI: Daily 11.5%  Incomplete SCI: Daily 4.7%  Wexner score 3.56*  Complete SCI: 5.3*  Incomplete SCI: 1.64* |  | Wexner scores were lower for individuals with injuries >T5 compared with lower injuries (p<0.01). Those with incomplete injuries had more bowel movements per week than those with complete injuries (p<0.0002).  Time to complete was longer in those with a longer duration of injury (p=0.0009). |
| **Mazor et al., 2016**  Knowles Questionnaire  FISI  [n=21] |  |  | **Incontinence:** FISI score 23±13  **Constipation:** Knowles score 19±4  71% report being constipated |  |  |
| **McCarthy et al., 2020**  NBD Score (modified): non-established  [n=50] | NBD Score: 20.6±8.8*  NBD Severity: 2% very minor; 8% minor; 10% moderate; 80% severe | **Frequency:** Daily 36%; 2-6 times weekly 58%; <once week 6%  **Times:** ≤30 min 22%; 31-60 min 44%; >60 min 34% | **Incontinence:** 100%*  < Monthly 40%; Monthly 26%; Weekly 26%; Daily 8% | Symptoms compatible with B-AD (uneasiness, headache, or perspiration during or after defecation) were reported by 34% of respondents. |  |
| **Nielsen et al., 2017**  Questionnaire: non-established  [n=109] |  | **Frequency:** <Every other day 10%  **Time:** ≥30 min 21% | **Incontinence:**  ≥Monthly 17%.  **Constipation:** 17%. |  | At the 19 year follow-up of the longitudinal study, more individuals considered themselves to be constipated (p<0.01) and to require >30 min to defecate (p<0.01) compared to the original visit. |
| **Noonan et al., 2008**  Questionnaire: non-established  [n=70] | Self-report bowel dysfunction 15% [n=69] |  |  |  |  |
| **Pardee et al., 2012**  Questionnaire: non-established  [n=241] |  | **Frequency:** Daily 43%  **Time:** >60 min 20% | **Incontinence [n=153]:** 43%  **Constipation [n=153]:** 44% |  |  |
| **Pires et al., 2018**  NBD score  [n=64] | NBD Severity: 33% very minor; 17% minor; 19% moderate; 31% severe | **Frequency:** Daily 9%; 2-6 times weekly 91%  **Time:** ≤30 min 50%; 31-60 min 47%; >60 min 3% | **Incontinence:** 100%*  <Monthly 75%; Monthly 16%; Weekly 6%; Daily 3% | Symptoms compatible with B-AD (uneasiness, sweating, or headaches during or after defecation) were reported by 13% of respondents. |  |
| **Stoffel et al., 2021** NBD score ADFSCI [n=1373] | NBD Score [n=1357]:12.0±6.0 NBD Severity [n=1357]: very minor/minor/moderate 58%; severe 42% |  |  | AD was reported by 40% of participants [n=1513]. ADFSCI Score: 7±4 Individuals with severe NBD reported higher ADFSCI scores than those with very minor to moderate NBD (8.0±4.4 vs. 6.8±4.1; p=0.001) On multivariable logistic regression, every point increase of the ADFSCI score was associated with 5% increased odds of having severe NBD symptoms (OR=1.05; p=0.02). | Experiencing AD, use of bowel medication or stimulation, and bladder management were significantly (p<0.05) associated with severe NBD symptoms (NBD>14). |
| **Ture et al., 2022**  NBD Score  [n=92] | NBD Score: 9.9±6.8  NBD Severity: 36% very minor; 13% minor; 23% moderate; 28% severe |  |  |  | AIS completeness was independently associated with moderate/ severe NBD. Individuals with complete SCI were more likely to develop moderate/ severe NBD than those with AIS C (OR=6.52; p=0.005) and those with AIS D (OR=17.2; p<0.001). |
| **Van Doorn et al., 2022**  NBD Score FISI  [n=55] | NBD Score [n=54]: 11.9±6.4 |  | **Incontinence [n=54]:** FISI score: 28±13 |  | Participants with higher FISI scores also had higher NBD scores (p<0.05) |
| **Westgren & Levi, 1998**  Questionnaire: non-established  [n=419] | Self-report bowel dysfunction 38% [n=320] |  |  |  |  |

*Abbreviations:* AD, autonomic dysreflexia, ADFSCI, autonomic dysfunction following spinal cord injury; B-AD, bowel care-induced autonomic dysreflexia; BCS; Bowel Continence Score; FI, faecal incontinence; FIM, Functional Independence Measure; FISI, Faecal Incontinence Severity Index; ISCIBDS International SCI Basic Dataset; NBD, Neurogenic Bowel Dysfunction; SCI, spinal cord injury.

**Supplemental Table 4** *Impact of bowel dysfunction on QoL post-SCI.* Where appropriate, data are reported as mean ± standard deviation, or proportion of respondents (%) identifying with the category. Significant (p<0.05) statistical associations are reported. * denotes data calculated based on metrics provided. Sample sizes are provided for the measure as a whole, and for specific measures as applicable in cases where not all participants completed every metric.

| **Study / Instrument:**  **(non-) established, specificity / [n]** | **General QoL / Life Restriction / Satisfaction** | **Physical / Mental / Emotional / Social Health-related QoL** | **Additional results** |
| --- | --- | --- | --- |
| **Adriaansen et al., 2016**  SCIM-III: established, generic  ISCIBDS-QoL (v1): established, condition specific  [n=282] | **QoL [n=261]:**  In standard multiple regressions, constipation showed an independent negative association with overall QoL (β=-0.131, p=0.023).  **Satisfaction [n=263]:**  Using Mann-Witney U tests, individuals with constipation were found to be more dissatisfied with life (p<0.05). Dissatisfaction for life did not significantly differ between individuals with and without FI. | **Physical [n=264]:**  Individuals with constipation are less satisfied with their physical health compared with those without (p<0.05). Those with FI are also less satisfied with their physical health compared to those without FI (p<0.05).  **Mental [n=262]:**  Individuals with constipation are less satisfied with their psychological health (p<0.01). In those with FI, dissatisfaction with psychological health did not achieve significance. |  |
| **Burns et al., 2015**  Interviews, ICF framework: established, generic  [n=19] | **Restriction:**  In the activity domain, participants felt that structured bowel programs impacted their diet (e.g., "I will eat only once a day to limit chances of involuntaries..."), imposed a barrier to spontaneity and daily schedule (e.g., "Before, I was free, I didn't think about going to the bathroom, but now I need all kinds of special arrangements"), and felt limited by the time requirements (e.g., "What bothers me most... is to lose an hour and a half in which I could be doing something else"). | **Physical:**  Relating to the bodily functions domain, participants identified challenges with the lack of predictability and fear of incontinence (e.g., "The bowel system can activate at any time during the day"), medical complications (e.g., "...Because of the hard stool, I have a fissure and sometimes there is blood at wiping"), and pain or discomfort (e.g., "There are nurses with whom it doesn't come out as well and I feel bloated all the time").  **Emotional:**  In the domain of personal factors, participants frequently expressed a clear negative impact of bowel function on emotional well-being, describing feelings of embarrassment, frustration, and stress (e.g., "When you have an accident in public, like at the gym where I go three times a week, it's embarrassing"). Interviewees also described frustrations with the loss of autonomy (e.g., "because the transfer is difficult, I try to do as much has possible by myself, but I eventually need help"), as well as strong emotional overlay with the physical experience of bowel function post-SCI (e.g., "When I run out of time and don't sit long enough on the toilet, I might get a stain like some liquid. You can smell it. I hate it.").  **Social:**  In the participation domain, the uncertainty associated with bowel function and FI impacted the pursuit of education and employment (e.g., "[worst case scenario is] having an involuntary while working"), relationships (e.g., "...If I got in a serious relationship, I would have to say that involuntaries will eventually happen"), social participation (e.g., "Incontinences limit my outings"), and travel (e.g., "the fact that [a bowel movement] can come any time during the day limits the possibility of taking long trips"). | In the environmental factors, participants were concerned with the cost and requirements (equipment, supplies, space) of bowel care, as well as the lack of appropriate and consistent assistance (e.g., "what is least helpful is always having different nurses, there is nothing worse."). |
| **Carlozzi et al., 2013**  Group interviews, ICF framework (modified): non-established, generic  [n=27] |  | *If B-AD was not resolved, well-being suffered:*  **Physical:** e.g., headaches: “it’s like your brain is trying to bust out of your skull and the worst pain I’ve ever had”  **Emotional:** e.g., fear and anxiety: “I just realized I could feel my mortality”  **Social:** e.g., community integration: “they’re afraid they’re gonna get out and, all of a sudden, you’re gonna have an episode” |  |
| **Coggrave et al., 2009**  Questionnaire: non-established, bowel care-specific  [n=1334] | **QoL:**  58% Bowel management is a problem  On a scale of 0 (least) -10 (worst) effect, the impact of bowel function on QoL was scored as 4.72±2.5*.  **Restriction [n=1275]:**  60% Fit their life around bowel care a little or a lot  35% Felt that their routines were not very flexible or not flexible at all | *Proportion (%) of respondents reporting a little or a lot*  **Social:**  43% Bowel management affects social life [n=1236]  24% Bowel management interferes with relationships [n=1583]  26% Bowel management stops them from working [n=1168]  42% Bowel management prevents staying away from home [n=1231] |  |
| **Coggrave et al., 2012**  Questionnaire: non-established, bowel care-specific  [n=92] | **QoL:**  On a scale of 0 (no problem at all) to 10 (worst possible), the mean ability to live with bowel dysfunction was 6.0±2.5*†, with a median of 3.  **Satisfaction:**  On a scale of 0 (very dissatisfied) to 10 (very satisfied), the mean level of satisfaction with bowel routines was 3.5±2.5*, with a median of 2.  **Restriction:**  On a scale of 1 (not at all) to 4 (a great deal), the mean level of life restriction due to bowel care was 22.0±1.0*, with a median of 4. |  |  |
| **Craven et al., 2012**  HUI3: established, generic  [n=357] | **QoL:**  Health status was markedly lower for individuals with moderate/ significant bowel dysfunction compared with those with no/ mild bowel dysfunction (0.17±0.24 vs. 0.29±0.27; p<0.0001) |  |  |
| **Elmelund et al., 2019**  ISCIBDS-QoL: Established, condition specific  [n=684] | *Rated on a scale of 0 (very dissatisfied) to 10 (very satisfied)*  **Satisfaction [n=247]:**  Participants without FI were more satisfied with life in general (score 8) than those who have daily-to-weekly FI (score 7; p<0.01), or monthly or less often FI (score 6; p<0.01). | *Rated on a scale of 0 (very dissatisfied) to 10 (very satisfied)*  **Physical [n=247]:**  There was no difference in the satisfaction with physical health between those without FI (score 5), daily-weekly FI (score 5), or monthly or less FI (score 4.5).  **Mental & Emotional [n=247]:**  Individuals without FI were also more satisfied with their psychological and emotional health (score 8) than those with FI monthly or less (score 6.5; p<0.05). |  |
| **Erdem et al., 2017**  SF-36: established, generic  PGA: established, generic  Direct question (QoL): non-established, bowel care-specific  [n=42] | **QoL:**  The total NBD score was determined to be significantly positively correlated with both the PGA score (r=0.91; p<0.001) and self-reported impact on QoL as assessed by a 5-point Likert scale (r=0.92; p<0.001). The raw data for the PGA and Likert scale were not reported. | **Physical:**  The NBD score was not significantly correlated with the physical component summary score (PCS) (r=-0.187; p=0.235). All subscales but the PH and RP achieved significance.  **Mental:**  NBD severity (NBD score) was negatively correlated with mental health QoL (SF-36 MCS) (r=-0.872; p<0.001), in which every domain within the MCS (MH, RE, VT, SF) was also independently significantly correlated with the NBD score (p<0.05). |  |
| **Gong et al., 2021**  SCI-QoL excretion-related dimensions: established, condition-specific  [n=101] | **QoL:**  Those with “very minor” NBD severity report better excretion-related QoL scores than those with minor, moderate, or severe NBD (p<0.001). | **Physical:**  SCI-QoL Bowel management difficulty score 24.0±10.7.  Individuals who experienced bowel accidents report lower excretion-related QoL scores compared to those who did not experience bowel accidents (p<0.001). |  |
| **Gorman et al., 2021**  SCI-QoL excretion-related dimensions: established, condition-specific  [n=49] | **QoL:**  SCI-QoL bowel management domain score: 50.8±8.6 |  | Those with incomplete injuries (48.6±7.4) were reported to score better on the SCI-QoL bowel management domain than those with complete injuries (52.1±9.1; p=0.172*), although this did not achieve statistical significance. |
| **Hicken et al., 2001**  SWLS: established, generic  CHART: established, generic  SF-12: established, generic  [n=106] | **Satisfaction:**  Compared with participants who were independent in their bowel management, those who were dependent reported lower SWLS (22.28±8.13 vs. 17.58±7.75; p<0.01) and CHART total scores (434.56±63.52 vs. 364.73±91.91; p<0.001). | **Physical:**  Compared with participants who were independent in their bowel management, those who were dependent reported lower scores in the CHART physical independence (93.25±17.86 vs. 67.80±28.67; p<0.001) and mobility (93.98±15.06 vs. 73.33±22.38; p<0.001) domains, as well as the physical health summary on the SF-12 (41.26±12.04 vs. 35.16±8.57; p<0.05).  **Emotional:**  There was no difference between those who were independent and dependent in their bowel management on perceived mental health in the SF-12 MCS (53.62±9.96 vs. 55.17±12.38; p>0.05).  **Social:**  There was no difference between those who were independent and dependent in their bowel management on the social integration domain of the CHART (90.61±16.43 vs. 86.31±21.31; p>0.05). |  |
| **Hwang et al., 2017**  SF-12v2: established, generic  SWLS: established, generic  CHART: established, generic  [n=131] | **QoL:**  SF-12 and SWLS scores did not change significantly in relation to the presence or absence of FI, or over time (p>0.05). | **Physical:**  The occurrence of FI was negatively associated with CHART mobility scores over time (β=-0.079; p=0.015). |  |
| **Inskip et al., 2018**  ISCIBDS (modified):  non-established, bowel care-specific  [n=287] | **QoL [n=219]:**  Impact of bowel care on QoL: Some impact 45%; Major impact 29%  78% Find bowel management is a problem  On a scale of 1 (least) -10 (worst) effect, the mean impact of bowel function on QoL was 5.9±2.8*, the mode was 10.  Time to complete current bowel routine was a significant predictor of QoL (p<0.001).  In multiple linear regressions, the severity of AD symptoms during bowel care emerges as a significant predictor of QoL (β=0.185, p=0.036).  **Restriction [n=214]:**  85% Fit their life around bowel management  **Satisfaction [n=237]:**  43% Are dissatisfied/ very dissatisfied with their normal bowel management routine | **Physical:**  *Percentage of individuals with B-AD (n=122) who find AD interferes with the following activities:*  51% Activities of daily living  51% Exercise  38% Rehabilitations  **Social:**  *Proportion (%) of respondents (n=214) reporting a little or a lot*  70% Felt bowel management interferes with their social life  62% Felt bowel management stops them from staying away from home  60% Felt bowel management interferes with personal relationships  41% Felt bowel management stops them from working outside the home  *Percentage of individuals with B-AD (n=122) who find AD interferes with the following activities:*  53% Social activities  50% Work  41% Sexual activity  35% Driving | Predictors of severity of AD symptoms during routine include the number of bowel management approaches used (p<0.001) and fatigue (p<0.001). |
| **Jorgensen et al., 2017**  SWLS: established, generic  SCIM-III: established, generic  [n=123] | **Satisfaction [n=111]:**  In multivariable linear regression, neither the presence of bowel problems including FI and constipation (β=0.6; p=0.63) nor nonvoluntary bowel function (β=-0.3; p=0.82) emerged as significant independent predictors of life satisfaction (total SWLS score). |  |  |
| **Jorgensen et al., 2021**  LiSAT-11: established, generic  [n=78] | **Satisfaction [n=72]:**  Individuals with bowel-related problems were less satisfied with life as a whole than those without bowel-related problems (78% vs. 51% dissatisfied; p=0.018). | **Physical:** Satisfaction with physical health was similar between those with and without bowel problems (86% vs. 68% dissatisfied; p>0.05).  **Mental:** Individuals with bowel problems were more dissatisfied with their mental health than those without bowel problems (57% vs. 22% dissatisfied; p=0.002).  **Social:** Individuals with bowel problems were less satisfied with their contacts with friends (68% vs. 29% dissatisfied; p=0.001) and family life (16% vs. 41% dissatisfied; p=0.048) compared to those without bowel problems. There was no difference in satisfaction with sexual life or partner relationships (86% vs. 85% dissatisfied; p>0.05). | More secondary health conditions (bowel problems, bladder problems, nociceptive pain, neuropathic pain, spasticity) were associated with less satisfaction with life as a whole, as well as social, physical, and mental health (p<0.05). |
| **Kannisto & Rintala, 1995**  Questionnaire: non-established, bowel care-specific  [n=35] |  | **Social:**  26% Felt fully satisfied with their bowel function and no limitations in their social life  69% Had some difficulties with their bowel function and minor social limitations due to bowel dysfunction  6% Had severe difficulties with their bowel function and marked social limitations |  |
| **Kim et al., 2012**  Questionnaire: non-established, bowel care-specific  [n=388] | **QoL:**  64% Report moderate to severe deterioration of general QoL due to problems with defecation.  In logistical regression modelling, symptoms of possible AD were not significantly associated with reduced general QoL .  Severe deterioration in generalized QoL was associated with >60 min time to complete (β=11.21; p=0.01). | **Social:**  67% Report moderate to severe deterioration of social QoL due to defecation  64% Report moderate to severe deterioration of home QoL due to defecation  Symptoms of possible AD were not associated with reduced social QoL (p>0.05).  Symptoms of possible AD contributed to severe deterioration in home QoL, but did not achieve statistical significance (β=1.73, p=0.07). | Frequency of FI had the greatest effects on QoL (general QoL β=67.4; p<0.01; social QoL β=47.69; p<0.01; home QoL β=26.87; p<0.01). |
| **Krogh et al., 1997**  Questionnaire: non-established, bowel care-specific  [n=424] | **Restriction:**  20% FI causes some or major restriction on QoL [n=395]  32% Disturbed defecation causes some or major restriction on QoL [n=413] | **Social [n=397]:**  39% report that colorectal dysfunction in general causes some or major restriction to social activities or QoL | Bowel dysfunction was a greater problem than both bladder and sexual dysfunction for 30% of respondents.  Women had a significantly higher risk of restriction on social activities caused by disturbed defecation (OR=1.83; p<0.02) and FI (OR=1.88; p<0.02) than men. |
| **Krogh et al., 2006**  Questionnaire: non-established, bowel care-specific  [n=424] | **QoL [n=355]:**  Impact of bowel problems on QoL: Major 16%; Some 21%; Minor 35%; No 28%  Severity of bowel dysfunction (as determined by the NBD score), is moderately correlated to impact on QoL (k=0.25). |  | Frequency of FI (OR=13.1; p<0.0001), time used for defecation (OR=6.8; p<0.0001), frequency of bowel movements (OR=6.1; p<0.0001), and digital stimulation or evacuation (OR=5.0; p<0.01) were most significantly associated with impact on QoL. |
| **Koyuncu et al., 2017**  SF-36: established, generic  [n=55] |  | **Physical:**  Physical health domains (GH, PF, RP, or BP) scores were not significantly correlated (p>0.05) with the severity of bowel dysfunction (NBD score).  **Mental:**  RE domain scores were weakly positively correlated with increasing severity of bowel dysfunction (NBD score) (r=0.185; p=0.007). No other significant relationships were determined between the SF-36 mental health domains and NBD scores. |  |
| **Leduc et al., 2002**  Questionnaire: non-established, bowel care-specific  [n=30] | **QoL:**  Decrease in QoL due to gastrointestinal disorder: Little to no 73%; Moderate to severe 27% |  | Individuals reporting moderate to severe decreases in QoL due to gastrointestinal disorders tended to have longer colonic transit time than those with little to no reduction in QoL, but the difference did not quite achieve significance (58.6±40.7hrs vs. 80.7±63.9hrs; p>0.05). |
| **Liu et al., 2009**  SF-36: established, generic  [n=128] |  | **Physical:**  Using general linear modeling, it was determined that physical health QoL (SF-36 PCS) decreased with increasing severity of bowel dysfunction (NBD score) (p<0.001). This was largely driven by the physical function domain in the PCS (p<0.001).  **Mental:**  Mental health QoL (SF-36 MCS) was not associated with NBD severity (p=0.18). | Individuals with tetraplegia (ASIA A, B, C) reported significantly lower PCS scores (p<0.001) compared with individuals with paraplegia (ASIA A, B, C), and individuals with ASIA D lesions of all levels. |
| **Locke et al., 2019**  LiSAT-11: Established, generic  SF-12: Established, generic  [n=1529] | **QoL:**  In multivariable analysis, constipation (OR=0.534; p<0.0001) was significantly associated with poor QoL scores. |  |  |
| **Lombardi et al., 2010**  SF-36: Established, generic  [n=23] |  | **Emotional:**  At baseline, the median RE domain scores were lower in individuals with chronic FI compared with those with chronic constipation (47.4 vs. 54.0).  **Social:**  The median SF domain score was lower in those with chronic FI than those with chronic constipation (49.1 vs. 58.2).  *Statistical comparison not provided* |  |
| **Luther et al., 2005**  IBDQ (modified): Established, bowel care-specific  [n=296] | **Satisfaction [n=271]:**  Satisfaction with bowel care program: Very satisfied 11%; Somewhat satisfied 18%; Somewhat dissatisfied 29%; Very dissatisfied 42% | *IBDQ score /7*  **Emotional:**  Troubled by unplanned bowel evacuations: 5.9±1.4  Felt socially embarrassed due to bowel problems: 6.1±1.5  **Social:**  Unable to attend work/ school or fulfill responsibilities due to bowel problems: 5.9±1.6  Delay or cancel social engagement due to bowel problems: 6.1±1.3  Difficulty doing leisure or sports activities due to bowel problems: 5.9±1.6  Bowel problems limit sexual activity: 6.1±1.9 |  |
| **Lynch et al., 2000**  Questionnaire: non-established, bowel care-specific  [n=467] | **QoL:**  62% Incontinence affects life to some degree  8% Incontinence always affects everyday life |  |  |
| **Mazor et al., 2016**  Questionnaire: non-established, bowel care-specific  [n=21] | *Rated on a scale of 1 (least) - 10 (most)*  **QoL:** Effect of bowel dysfunction on QoL 7.2±2.4  **Satisfaction:** Satisfaction with bowel movements 2.8±2.2 |  | *Rated on a scale of 1 (least) - 10 (most)*  Control over bowel movements 2.8±1.9 |
| **McCarthy et al., 2020**  Questionnaire: non-established, bowel care-specific  [n=50] | **QoL:**  On a scale of 0 (best) to 5 (worst), the mean impact of bowel management on QoL was 4.0.  **Satisfaction:**  On a scale of 0 (worst) to 10 (best), the mean general satisfaction with bowel management was 3.2±2.5* |  |  |
| **Nevedal et al., 2016**  Interviews, ICF framework: established, generic  [n=50] | **Restriction:**  In the activity domain, interviewees found life was controlled by bladder and bowel medical management. In the personal factors domain, interviewees explained the importance of redefining and finding independence. | **Emotional:**  In the bodily functions domain, participants reported worrying about the potential for accidents (FI) when leaving the home (e.g., "I'm afraid to go anywhere and do anything because my bowels are going to work up and that embarrasses me. I don't want to be embarrassed or embarrass my husband or mess up my car or his car, or stuff like that"), and struggling with the vulnerability and frustration of relying on others to clean up after accidents.  **Social:**  In the participation domain, meta-themes that emerged were that life was controlled by their bladder and bowel (e.g., "It's really hard to have your life controlled by your bladder and bowel function"), and life course disruption as interviewees lamented the abrupt loss of independence after SCI (e.g., "Like a baby, completely dependent on somebody to change your diaper. When a woman finds herself wearing diapers, it's a very difficult thing"). |  |
| **Nielsen et al., 2017**  Questionnaire: non-established, bowel care-specific  [n=109] | *Proportion (%) of respondents reporting some or major restriction*  **Restriction**  26% Colorectal dysfunction restricts general condition  25% Difficult bowel evacuation restricts QoL  16% Constipation causes restrictions on QoL  15% FI causes restriction on QoL |  |  |
| **Noonan et al., 2008**  SF-36: Established, generic  QoL numeric rating: non-established, generic  [n=70] | **QoL:**  The presence of bowel, bladder, and sexual dysfunction did not significantly reduce QoL (p=0.79).  **Satisfaction:**  The presence of bowel, bladder, and sexual dysfunction did not significantly reduce the odds of satisfaction with living with the current symptoms (p=0.54). | **Physical:**  The presence of bowel, bladder, and sexual dysfunction was found to be a strong predictor of reduced physical QoL (SF-36 PCS; β=-6.9, CI -11.6 to -2.2; p=0.004).  **Mental:** Mental health QoL (SF-36 MCS) did not differ between those with and without bowel, bladder, and sexual dysfunction (β=1.4; p=0.65). |  |
| **Pardee et al., 2012**  Burwood questionnaire: established, bowel care-specific  Direct question (satisfaction): non-established, bowel care-specific  [n=241] | **QoL:**  15% indicate AD is a problem in relation to their bowel program  **Satisfaction [n=235]:**  Satisfaction with bowel management: Satisfied 54%; Neutral 23%; Dissatisfied 23%  Chi-squared analysis revealed no significant differences in the level of satisfaction with their bowel program between those who did and did not report AD as a problem with their bowel program (p>0.05). | *Proportion (%) of respondents reporting sometimes, often, or always*  **Emotional [n=234]:**  59% Troubled by bowel function  29% Troubled by fear of not finding bathroom  40% Felt irritable because of bowel problems  40% Felt lack of understanding about bowel function from others  **Social:**  **[n=245]**  25% Avoid attending events with no close bathroom  47% Difficulty doing leisure or sports  42% Delay or cancel social engagement because of bowel problems  28% Bowel problems limit sexual function  **[n=236]**  12% Unable to attend work because of bowel problems  27% Feel ability to work to their own standards are limited by bowel function  17% Work standards slip because of bowel habit  87% Happy with work ethic and achievements | Individuals who indicated satisfaction with their current bowel program reported better QoL on the Burwood symptom (p=0.001), bowel problem (p<0.001), work function (p<0.021), and social function (p<0.001) subscales.  Individuals who were dissatisfied with their bowel program indicated problems of time (p=0.001), pain or discomfort (p=0.033), and poor results (p<0.001).  25% of individuals with SCI with ostomies for bowel management cite AD as the reason for getting the ostomy. |
| **Pires et al., 2018**  Questionnaire: non-established, bowel care-specific  [n=64] | **QoL:**  59% Report a negative impact of NBD on QoL  Moderate and severe NBD was strongly associated with patients’ self-reported QoL (OR=45.0; p<0.01). | **Emotional:** Moderate and severe NBD was strongly associated with feelings of frustration, anxiety, or depression (OR=25.0; p<0.01) |  |
| **Smith & Decter, 2015**  FIQL: established, bowel care-specific  [n=17] |  | *FIQL domain scores /4*  **Mental:** Depression and self-perception 2.8±0.9  **Emotional:** Embarrassment 2.2±1.1  **Social:** Lifestyle 2.3±0.9; Coping/ behavior 2.2±0.9 |  |
| **Stoffel et al., 2021**  SF-12: established, generic  [n=1373] |  | **Physical:**  PCS scores of those with severe NBD (39.6±10.8) were lower than those with very minor to moderate NBD (41.2±11.3; p=0.007).  **Mental:**  MCS scores of those with severe NBD (46.5±11.5) were lower than those with very minor to moderate NBD (49.5±11.1; p<0.0001). |  |
| **Sweet et al., 2014**  LiSAT-11: established, generic  Conceptual structural equation model: non-established, generic [n=1137] | **QoL:**  In the conceptual model, bowel and bladder complications were indirectly related to reduced QoL; bowel and bladder complications were significantly related to unmet vital needs (housing, equipment, transportation; β=0.56; p<0.05), which was significantly associated with QoL (β=-0.22; p<0.05). |  |  |
| **Ture et al., 2022** SF-36: established, generic FIM: established, generic  [n=92] |  | **Physical:** Compared to those with very minor NBD, individuals with moderate and severe NBD had lower motor independence (FIM; p=0.009) and lower physical function QoL (SF-36 PH; p<0.05).  **Mental:** No significant relationships were identified between the SF-36 mental health domains and NBD severity groups (p>0.05). |  |
| **Van Doorn et al., 2022**  EQ-5D-3L: established, generic FIQL: established, bowel care-specific  [n=55] | **QoL:** FIQL Total: 3.2±0.8  Participants with lower FIQL and EQ-5D-3L scores had significantly higher NBD scores (both p<0.001). | *FIQL domain scores /4*  **Mental:** Depression and self-perception 3.4±0.9  **Emotional:** Embarrassment 3.0±1.0 **Social:** Lifestyle 3.3±0.9; Coping/ behaviour 3.1±0.9 |  |
| **Westgren & Levi, 1998**  SF-36: established, condition specific  [n=320] |  | **Physical:**  Comparing individuals with and without bowel problems, those with bowel problems scored significantly lower in the PF, BP, and GH domains (p<0.005) but the RP domain scores remained comparable (p=0.075).  **Mental:**  Individuals with bowel problems scored significantly lower in the VT, SF, RE, and MH domains of the SF-36 (p<0.005). | Individuals with tetraplegia scored markedly lower on the physical function domain compared with individuals with paraplegia (p<0.001) while all other domain scores remained comparable.  Individuals with full time employment scored better in all domains compared to individuals without full-time employment (p<0.005). |

*Abbreviations:* B-AD, bowel care-induced autonomic dysreflexia; CHART, Craig Handicap Assessment and Reporting Technique; FI, faecal incontinence; FIQL, Faecal Incontinence QoL; HUI3, Health Utilities Index Mark III; IBDQ; Inflammatory Bowel Disease Questionnaire; ICF, International Classification of Functioning, Disability, and Health; ISCIDBS, International SCI Basic Data Set; LiSAT-11, Satisfaction with Life questionnaire; PGA, Physician Global Assessment; SCIM-III, Spinal Cord Independence Measure III; SF-12, 12-item Short Form health survey; SF-12v2 (physical functioning, PF; role limitations due to physical health, RP; bodily pain, BP; general health, GH; vitality, VT; social functioning, SF; role limitations due to emotional health, RE; mental health, MH), 12-item Short Form health survey version 2; SF-36, 36-item Short Form health survey; SWLS, Satisfaction with Life Scale

**SUPPLEMENTARY REFERENCES**

1. Hubli M, Gee CM, Krassioukov A V. Refined assessment of blood pressure instability after spinal cord injury. Am J Hypertens [Internet]. 2015 Feb 1 [cited 2022 Nov 22];28(2):173–81. Available from: https://pubmed.ncbi.nlm.nih.gov/24990527/

2. Stoffel JT, Barboglio-Romo P, Lenherr SM, Elliott SP, O’Dell D, Myers JB, et al. Factors impacting bowel symptoms in a contemporary spinal cord injury cohort: results from the Neurogenic Bladder Research Group Registry. Spinal Cord [Internet]. 2021 Sep 1 [cited 2022 Sep 19];59(9):997–1002. Available from: https://pubmed.ncbi.nlm.nih.gov/34345004/

3. Holschneider A. Electromanometrie des Enddarmes. 1983.

4. Pardee C, Bricker D, Rundquist J, MacRae C, Tebben C. Characteristics of neurogenic bowel in spinal cord injury and perceived quality of life. Rehabil Nurs [Internet]. 2012 May [cited 2022 Aug 22];37(3):128–35. Available from: https://pubmed.ncbi.nlm.nih.gov/22549630/

5. Randell N, Lynch AC, Anthony A, Dobbs BR, Roake JA, Frizelle FA. Does a colostomy alter quality of life in patients with spinal cord injury? A controlled study. Spinal Cord [Internet]. 2001 [cited 2022 Jan 3];39(5):279–82. Available from: https://pubmed.ncbi.nlm.nih.gov/11438845/

6. Hall KM, Dijkers M, Whiteneck G, Brooks CA, Krause JS. The Craig Handicap Assessment and Reporting Technique (CHART): Metric Properties and Scoring. Top Spinal Cord Inj Rehabil [Internet]. 1998 Jul 1 [cited 2023 Jan 3];4(1):16–30. Available from: https://meridian.allenpress.com/tscir/article/4/1/16/85658/The-Craig-Handicap-Assessment-and-Reporting

7. Rabin R, De Charro F. EQ-5D: a measure of health status from the EuroQol Group. Ann Med [Internet]. 2001 [cited 2022 Nov 24];33(5):337–43. Available from: https://pubmed.ncbi.nlm.nih.gov/11491192/

8. Rockwood TH, Church JM, Fleshman JW, Kane RL, Mavrantonis C, Thorson AG, et al. Fecal Incontinence Quality of Life Scale: quality of life instrument for patients with fecal incontinence. Dis Colon Rectum [Internet]. 2000 Jan [cited 2022 Sep 7];43(1):9–16. Available from: https://pubmed.ncbi.nlm.nih.gov/10813117/

9. Rockwood TH, Church JM, Fleshman JW, Kane RL, Mavrantonis C, Thorson AG, et al. Patient and surgeon ranking of the severity of symptoms associated with fecal incontinence: the fecal incontinence severity index. Dis Colon Rectum [Internet]. 1999 [cited 2022 Sep 7];42(12):1525–31. Available from: https://pubmed.ncbi.nlm.nih.gov/10613469/

10. Granger C V, Hamilton BB, Keith RA, Zielezny M, Sherwin FS. Advances in functional assessment for medical rehabilitation. Top Geriatr Rehabil. 1986;1(3):59–74.

11. Leduc BE, Spacek E, Lepage Y. Colonic transit time after spinal cord injury: any clinical significance? J Spinal Cord Med [Internet]. 2002 [cited 2022 Aug 22];25(3):161–6. Available from: https://pubmed.ncbi.nlm.nih.gov/12214902/

12. Feeny D, Furlong W, Torrance GW, Goldsmith CH, Zhu Z, DePauw S, et al. Multiattribute and single-attribute utility functions for the health utilities index mark 3 system. Med Care [Internet]. 2002 Feb [cited 2022 Sep 7];40(2):113–28. Available from: https://pubmed.ncbi.nlm.nih.gov/11802084/

13. Pallis AG, Mouzas IA, Vlachonikolis IG. The inflammatory bowel disease questionnaire: a review of its national validation studies. Inflamm Bowel Dis [Internet]. 2004 May [cited 2022 Sep 7];10(3):261–9. Available from: https://pubmed.ncbi.nlm.nih.gov/15290922/

14. Luther SL, Nelson AL, Harrow JJ, Chen F, Goetz LL. A comparison of patient outcomes and quality of life in persons with neurogenic bowel: Standard bowel care program vs colostomy. J Spinal Cord Med. 2005;28(5):387–93.

15. Towards a Common Language for Functioning, Disability and Health ICF Towards a Common Language for Functioning, Disability and Health: ICF The International Classification of Functioning, Disability and Health [Internet]. Geneva ; 2002 [cited 2022 Aug 3]. Available from: https://cdn.who.int/media/docs/default-source/classification/icf/icfbeginnersguide.pdf?sfvrsn=eead63d3_4&download=true

16. Krogh K, Emmanuel A, Perrouin-Verbe B, Korsten MA, Mulcahey MJ, Biering-Sørensen F. International spinal cord injury bowel function basic data set (Version 2.0). Spinal Cord [Internet]. 2017 Jul 1 [cited 2022 Sep 7];55(7):692–8. Available from: http://dx.doi.org/10.1038/sc.2016.189

17. Inskip JA, Lucci VEM, McGrath MS, Willms R, Claydon VE. A Community Perspective on Bowel Management and Quality of Life after Spinal Cord Injury: The Influence of Autonomic Dysreflexia. J Neurotrauma [Internet]. 2018 May 1 [cited 2021 Sep 3];35(9):1091–105. Available from: https://pubmed-ncbi-nlm-nih-gov.proxy.lib.sfu.ca/29239268/

18. Charlifue S, Post MW, Biering-Sørensen F, Catz A, Dijkers M, Geyh S, et al. International Spinal Cord Injury Quality of Life Basic Data Set. Spinal Cord [Internet]. 2012 Sep [cited 2022 Aug 23];50(9):672–5. Available from: https://pubmed.ncbi.nlm.nih.gov/22450884/

19. Knowles CH, Eccersley AJ, Scott SM, Walker SM, Reeves B, Lunniss PJ. Linear discriminant analysis of symptoms in patients with chronic constipation: validation of a new scoring system (KESS). Dis Colon Rectum [Internet]. 2000 [cited 2022 Sep 7];43(10):1419–26. Available from: https://pubmed.ncbi.nlm.nih.gov/11052520/

20. Krogh K, Christensen P, Sabroe S, Laurberg S. Neurogenic bowel dysfunction score. Spinal Cord. 2006;44(10):625–31.

21. Pascoe VL, Enamandram M, Corey KC, Cheng CE, Javorsky EJ, Sung SM, et al. Using the Physician Global Assessment in a Clinical Setting to Measure and Track Patient Outcomes. JAMA Dermatology [Internet]. 2015 Apr 1 [cited 2022 Aug 3];151(4):375–81. Available from: https://jamanetwork.com/journals/jamadermatology/fullarticle/2039085

22. Melin R, Fugl-Meyer KS, Fugl-Meyer AR. Life satisfaction in 18- to 64-year-old Swedes: in relation to education, employment situation, health and physical activity. J Rehabil Med [Internet]. 2003 Mar [cited 2022 Sep 7];35(2):84–90. Available from: https://pubmed.ncbi.nlm.nih.gov/12691338/

23. Tulsky DS, Kisala PA. The Spinal Cord Injury – Quality of Life (SCI-QOL) measurement system: Development, psychometrics, and item bank calibration. J Spinal Cord Med [Internet]. 2015 May 1 [cited 2022 Sep 7];38(3):251. Available from: /pmc/articles/PMC4445017/

24. Ware JE, Kosinski M, Keller SD. SF-36 Physical and Mental Health Summary Scales: a User’s Manual Estimation of medical care total expenditures View project [Internet]. Health assessment Lab. Boston; 1994 Dec [cited 2022 Nov 24]. Available from: https://www.researchgate.net/publication/292390260

25. Ware JE, Kosinski M, Keller SD. A 12-Item Short-Form Health Survey: construction of scales and preliminary tests of reliability and validity. Med Care [Internet]. 1996 [cited 2022 Sep 7];34(3):220–33. Available from: https://pubmed.ncbi.nlm.nih.gov/8628042/

26. Montazeri A, Vahdaninia M, Mousavi SJ, Asadi-Lari M, Omidvari S, Tavousi M. The 12-item medical outcomes study short form health survey version 2.0 (SF-12v2): a population-based validation study from Tehran, Iran. Health Qual Life Outcomes [Internet]. 2011 Mar 7 [cited 2022 Nov 24];9. Available from: https://pubmed.ncbi.nlm.nih.gov/21385359/

27. Ware JE, Sherbourne CD. The MOS 36-item short-form health survey (SF-36). I. Conceptual framework and item selection. Med Care. 1992;30(6):473–83.

28. Jorge JM, Wexner SD. Etiology and management of fecal incontinence. Dis Colon Rectum [Internet]. 1993 Jan [cited 2022 Sep 7];36(1):77–97. Available from: https://journals.lww.com/dcrjournal/Fulltext/1993/36010/Etiology_and_management_of_fecal_incontinence.16.aspx
